# Global Patterns of Mercury Speciation and Biomagnification in Sharks: Ecological Drivers and Food Safety Implications

**DOI:** 10.1021/acs.est.6c01257

**Published:** 2026-05-08

**Authors:** Ginevra Boldrocchi, Davide Spanu, Alejandro Ruiz López, Fulvio Garibaldi, Luca Lanteri, Alberto Maggi, Roberta Bettinetti, Damiano Monticelli

**Affiliations:** † Department of Human Sciences, Innovation and Territory, 19045 University of Insubria, Via Valleggio 11, 22100 Como, Italy; ‡ One Ocean Foundation, Via Gesù 10, 20121 Milan, Italy; § Department of Science and High Technology, 19045 University of Insubria, Via Valleggio 11, 22100 Como, Italy; ∥ Department for Earth, Environment and Life Sciences, University of Genoa, Corso Europa 26, 16132 Genoa, Italy

**Keywords:** elasmobranchs, heavy metals, food safety, shark consumption

## Abstract

Mercury (Hg) is a global contaminant that biomagnifies in food webs, raising concerns for food safety, fisheries exploitation, and wildlife conservation. Fish, including apex predators like sharks, are the primary source of human Hg exposure, yet species-specific speciation data remain scarce. Most studies rely on total Hg (THg) as a proxy for methylmercury (MeHg), but direct MeHg measurement is essential for accurate risk assessment due to neurotoxicity and bioavailability. This study presents a comprehensive assessment, quantifying THg-MeHg in 18 species from the Mediterranean, Indian, and Atlantic Oceans, nine measured for the first time. Concentrations varied widely, with deep-sea and pelagic sharks showing highest levels. THg and MeHg strongly correlated (R^2^ = 0.99), but MeHg-THg ranged 65–101%, demonstrating substantial interspecific variability and challenging the assumption of near-complete methylation. Bioaccumulation increased with body size and trophic level, and biomagnification was pronounced in Mediterranean deep-sea assemblages. Nearly half of the species exceeded the 1 mg kg^–1^-EU Hg limit. Target Hazard Quotients exceeded 1 for deep-sea and large pelagic sharks, highlighting tangible health risks. Elevated MeHg levels in commercial fillets confirm consumer exposure. Species with the highest MeHg burdens are heavily exploited and threatened, identifying globally traded sharks as hotspots of human Hg exposure.

## Graphical abstract

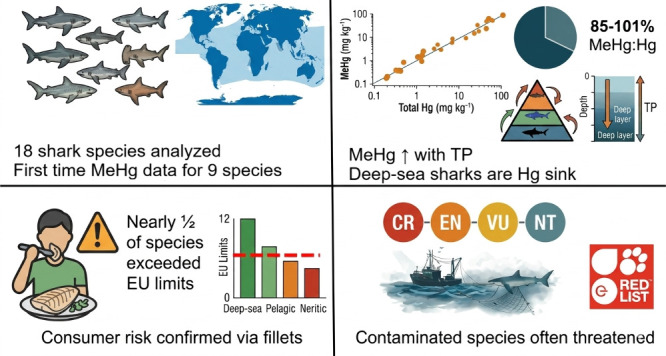

## 1 Introduction

Mercury (Hg) is a pervasive global contaminant whose anthropogenic emissions have substantially increased its mobility and bioavailability in aquatic ecosystems.^1–3^ Microbial methylation converts inorganic Hg into methylmercury (MeHg), a potent neurotoxical agent that bioaccumulates efficiently (bioconcentration factors ∼10^4^–10^7^) and biomagnifies across food webs, resulting in the highest burdens in top predators.^4,5^ Accordingly, the proportion of MeHg relative to total Hg (THg) increases with trophic level, from ∼5% in seawater to nearly 100% in piscivorous species fish.^6–8^ Human exposure occurs primarily through seafood consumption, with well-documented developmental and systemic health effects.^9,10^

Methylmercury exposure also induces adverse effects in fish and other aquatic organisms, including impaired reproduction, altered behavior, neurological damage and mortality.^11–13^ These effects are particularly relevant for long-lived, high trophic level predators such as sharks, which efficiently accumulate Hg,^14–18^ Many shark species are heavily exploited worldwide,^19^ increasing the relevance of these contaminants for food safety.

While multiple studies have quantified THg in sharks, a critical knowledge gap persists regarding the direct quantification of MeHg, the most toxicologically relevant form, across the broad biodiversity and biogeographic regions of these species.^20^ Indeed, most studies assume near-complete methylation of Hg in shark muscle,^21–25^ yet evidence indicates that MeHg-THg ratios vary with multiple biological and ecological factors, including species identity, body size, trophic position, and habitat use.^16,26–28^ This raises fundamental questions about the reliability of THg as a universal proxy for MeHg across elasmobranch taxa.^29^

This knowledge gap is particularly relevant given that sharks constitute a vital protein source and an expanding component of the global seafood trade, especially in regions with high per-capita consumption.^19^ Many harvested species are top predators, which are known to accumulate high levels of MeHg, raising concerns over human exposure. At the same time, several of these species are threatened with extinction, highlighting the broader conservation implications of their exploitation. Understanding species-specific Hg speciation in sharks therefore lies at the intersection of public health, food safety, and conservation priorities. However, a coordinated evaluation of Hg speciation across multiple shark species and ocean basins is lacking.

Thus, the present study provides a multiocean assessment of THg and MeHg in a large and taxonomically diverse assemblage of shark species (N = 18), from the Mediterranean Sea, Indian and Atlantic Oceans, including nine species for which MeHg is quantified for the first time. This represents the first systematic effort to directly evaluate Hg speciation across such a broad range of shark taxa and ecological niches. Specifically, THg and MeHg concentrations and MeHg-THg ratios were determined to assess inter- and intraspecific variability in relation to sex, body size, trophic position, and geographic origin.

Additionally, to place our findings within the context of existing knowledge, a targeted literature review was conducted to identify gaps in species coverage and quantify how rarely MeHg has been directly measured in sharks, despite its central role in toxicity and risk assessment. The reliability of THg as a proxy for MeHg was also examined, and potential human health risks associated with shark meat consumption were quantified.

By identifying species that are both highly contaminated and at elevated risk of extinction, these results reveal a clear convergence between food-safety and conservation concerns. These findings have direct implications for environmental monitoring, fisheries management, and public health, and support the need to discourage consumption of shark species posing the greatest risk of MeHg exposure.

## 2 Materials and Methods

### 2.1 Sample Collection

Muscle samples of *Carcharhinus macloti*, *C. melanopterus*, *C. sorrah*, *Hemigaleus microstoma*, *Mustelus mosis*, *Rhizoprionodon acutus*, *Sphyrna lewini*, and *Stegostoma fasciatum* were obtained from fish markets in Djibouti City (2017–2021).^26^ *Cetorhinus maximus* samples from the Mediterranean Sea were obtained from individuals already deceased as fisheries bycatch in trammel nets and landed in Liguria, as previously reported by Boldrocchi et al. (2022).^30^ Additional samples (*Dalatias licha*, *Etmopterus spinax*, *Galeorhinus galeus*, *Galeus melastomus*, *Heptranchias perlo*, *Hexanchus griseus*) were collected in 2021–2022 from the Mediterranean Sea from local fish markets in Liguria, while fillets of *Prionace glauca*, *Isurus oxyrinchus*, and *Squalus acanthias* from the Atlantic Ocean (FAO 47–34 and 27) were purchased in Milan, Italy (2022) from retail suppliers. For these latter commercial samples, species identification was based on labeling information compliant with EU traceability regulations.

Ethical approval was not required for this study in accordance with local legislation and institutional requirements. This study did not involve live animal experimentation or the handling of live specimens. All samples consisted exclusively of post-mortem tissues obtained from commercially available fish products (fish markets, fishermen, and retail suppliers) or from fisheries bycatch specimens already dead at the time of collection.

Biological data (total length, weight, sex) were recorded when available. Following two studies,^31,32^ trophic position was assigned using FishBase^33^ to ensure a standardized and comparable metric across species, given the variability of literature-derived estimates based on different methodologies and ecosystems, as previously adopted (e.g., Roff et al.^31^). Ecological classifications (neritic, pelagic, deep-sea) and conservation status were obtained from the 2025 IUCN Red List (Supporting Information, Table S1).

The methodology used for the systematic collection and validation of published Hg and MeHg data in elasmobranchs is described in the Supporting Information under “Additional Methodological Details”.

### 2.2 Mercury and Methylmercury Determination

Total Hg was quantified using an inductively coupled plasma–mass spectrometry system (iCAP Q ICP-MS, Thermo Scientific) after microwave-assisted digestion carried out with a Milestone ETHOS One (dissolved mass approximately 20 mg). For digestion, a multibatch setup (see our previous work^34^ was employed using 0.5 mL of high-purity HNO_3_ and 0.5 mL of high-purity HCl, both obtained through sub-boiling distillation). The digestion protocol consisted of a simple heating program: a 20 min ramp from room temperature to 110 °C, followed by a 30 min hold at 110 °C.

Methylmercury extraction and selective quantification followed the procedure described in earlier work. In summary, approximately 20 mg of each sample was combined with 5 mL of an extracting solution containing 0.5 M HCl, 3.3 mM thiourea, and 0.037 M HBr in a 10 mL polypropylene tube. The mixture was sonicated for 15 min at room temperature using a Branson 5800 ultrasonic bath. After sonication, the suspensions were centrifuged at 4000 rpm for 10 min using an ALC 4206 instrument. The resulting solutions were analyzed for MeHg by installing a short column—20 mm in length and 2.5 mm internal diameter—packed with a strong anion-exchange resin between the peristaltic pump and the ICP-MS nebulizer.^35^

Quality control was ensured by analyzing procedural blanks and certified reference materials within each analytical batch. For THg determination, the certified fish muscle reference material ERM-BB422 (Institute for Reference Materials and Measurements, Geel, Belgium) was included, yielding a mean recovery of 109 ± 9% (n = 7; uncertainty reported as one standard deviation). Quality assurance for MeHg analysis was evaluated using the tuna fish muscle reference material CRM-463 (European Commission, Community Bureau of Reference), which produced an average recovery of 102 ± 6% across six replicate analyses (uncertainty corresponding to one standard deviation).

### 2.3 Trophic Magnification Factor

The Trophic Magnification Factor (TMF) estimations are based on the linear regression between log transformed contaminant concentrations (log_10_[C]) and trophic positions (TP). The TMF was calculated as 10^b^, with “b” being the slope of the following linear regression equation:

$$\log_{10}\left[C\right]=a+b\,\times TP$$

TMF > 1 indicates biomagnification along the food web; TMF < 1 indicates decreasing concentration with trophic level. TMF was calculated for the Mediterranean assemblage only.

### 2.4 Human Health Risk Assessment

MeHg exposure via shark consumption was assessed using the Target Hazard Quotient (THQ) and provisional tolerable weekly intake (PTWI), developed by the United States Environmental Protection Agency. THQ was calculated using the following equation:

$$THQ=\frac{Ef\times Ed\times FIR\times C}{BW\times AT\times RfD}\times 10^{-3}$$

where Ef is the exposure frequency (worst scenario: 365 days year^–1^; and 48 days year^–1^ considering fish consumption once a week); Ed is the exposure duration (83 years equivalent to average lifetime for Italian adults according to Italian National Institute of Statistics); FIR is the daily fish consumption in Italy (79.5 g day^–1^ according to FAO); C is the concentration of MeHg in the food (mg kg^–1^ ww), RfD is the oral reference dose of the USEPA (MeHg: 0.0001 μg g^–1^ day^–1^), BW is the average body weight (70 kg for an adult), and AT is the averaging exposure time for noncarcinogens (365 year^–1^ × exposure duration). The THQ functions as a ratio where if THQ > 1, adverse health effects are possible and if THQ < 1, the product is safe for consumption without expecting noncarcinogenic adverse health effects.^36^

Estimated weekly intake (EWI) was calculated as

$$\mathrm{EWI}=\frac{MeHg\times IR}{BW}$$

where MeHg represents the average MeHg concentration (mg kg^–1^ ww) in each shark species measured in this study, IR is weekly ingestion rate for total population (271.6 g weekly^–1^;^37^ and BW is the body weight. The estimated weekly intakes (EWI) were then compared to the Provisional Tolerable Weekly Intake (PTWI) of 1.6 μg MeHg kg^–1^ human body weight established by the joint FAO/WHO expert committee on Food Additives to ensure protection from developmental neurotoxicity in humans.^38^

Following Bezerra et al.,^39^ the number of meals containing the studied shark species was also determined based on the maximum meal frequency (CR_lim_) for each species for noncarcinogenic effects, using the following equation:

$${CR}_{\lim}=\frac{BW\times RfD}{C}$$

Daily consumption limitations can be expressed as the acceptable number of fish meals that can be consumed within a specific time period.^40^ Thus, the CR_lim_ (kg day^–1^) was converted to determine the allowable monthly meals for each studied species without risks of deleterious effects on human health:

$$\mathrm{CR}\mathrm{mm}=\frac{CR\max\times Tap}{MS}$$

where Tap represents the average number of days in months (30.44 days month-^1^), and MS the average size of a meal, which was established as 0.227 kg for adults.^36^ The US EPA recommends a threshold of 16 monthly meal of contaminated fish to avoid incurring in noncarcinogenic risk.

Since threshold values defined by regulatory guidelines of health agencies are established in wet weight (ww), dry weight data were converted to ww (∼70% moisture).^41,42^

### 2.6 Statistical Analyses

The significance level was set to α = 0.05. Normality and homogeneity were assessed using Shapiro–Wilk and Levene’s tests. THg, MeHg, and %MeHg differences among species were tested by one-way ANOVA, after a square root transformation to meet both assumptions of normality and homogeneity, with post hoc Tukey’s method.

Reproduction type effects in females were assessed via Kruskal–Wallis and Steel–Dwass tests. Independent *t* tests compared Hg metrics between sexes. Linear regressions evaluated relationships between Hg, MeHg, and shark length. PCA was performed using CAT (Chemometric Agile Tool) with autoscaling and varimax rotation. Data were autoscaled and the varimax rotation was applied (https://gruppochemiometria.it/index.php/software).

## 3 Results and Discussion

### 3.1 Global Speciation and Urgency of MeHg Coverage

A critical review of published literature on Hg in shark muscle reveals a substantial lack of MeHg speciation data across global populations. From 1975 to 2025, a total of 139 published articles on Hg pollution in shark muscles, explicitly reporting species or taxonomic order, were identified (Supporting Information, Table S2). Only 18.7% (N = 26) of these studies analyzed Hg speciation in sharks, while the remaining either assumed all Hg in muscle was MeHg, did not distinguish Hg forms, or analyzed only MeHg without THg.

Globally, prior to this study, the MeHg-to-THg ratio has been investigated in only 43 species, representing approximately 8% of shark biodiversity (N = 536 species^43^). While scientific attention toward Hg in sharks has increased markedly since 1980s (1975–2025; R^2^ = 0.56, n = 139, *p* < 0.0001) (Supporting Information, Figure S1), the growth in MeHg studies was less pronounced, although a weaker but still significant upward trend was also found (1975–2025; R^2^ = 0.14, n = 29, p = 0.0355).

The present study adds data for nine shark species analyzed for the first time, increasing the coverage of species-specific MeHg data to nearly 10% of known shark biodiversity. Given the limited MeHg data across ecologically and commercially relevant taxa, these results highlight the urgent need for broader Hg speciation studies. Direct MeHg quantification remains essential, as THg cannot reliably predict MeHg levels for accurate risk assessment in both conservation and food-safety contexts.^29^

### 3.2 THg and MeHg among Shark Species

A total of 63 individual elasmobranchs representing 18 species were analyzed for both THg and MeHg, with species-specific sample sizes ranging from 1 to 10 individuals (Supporting Information, Tables S1 and S3). Principal Component Analysis (PCA) using THg, MeHg, trophic position (TP), and total length (TL) revealed a clear structure in the data set: PC1 (50% variance) was driven by THg and MeHg, representing bioaccumulation, while PC2 (37% variance) captured trophic-related variation, including MeHg-THg ratios, reflecting biomagnification processes (Figure 1).

**Figure 1:**
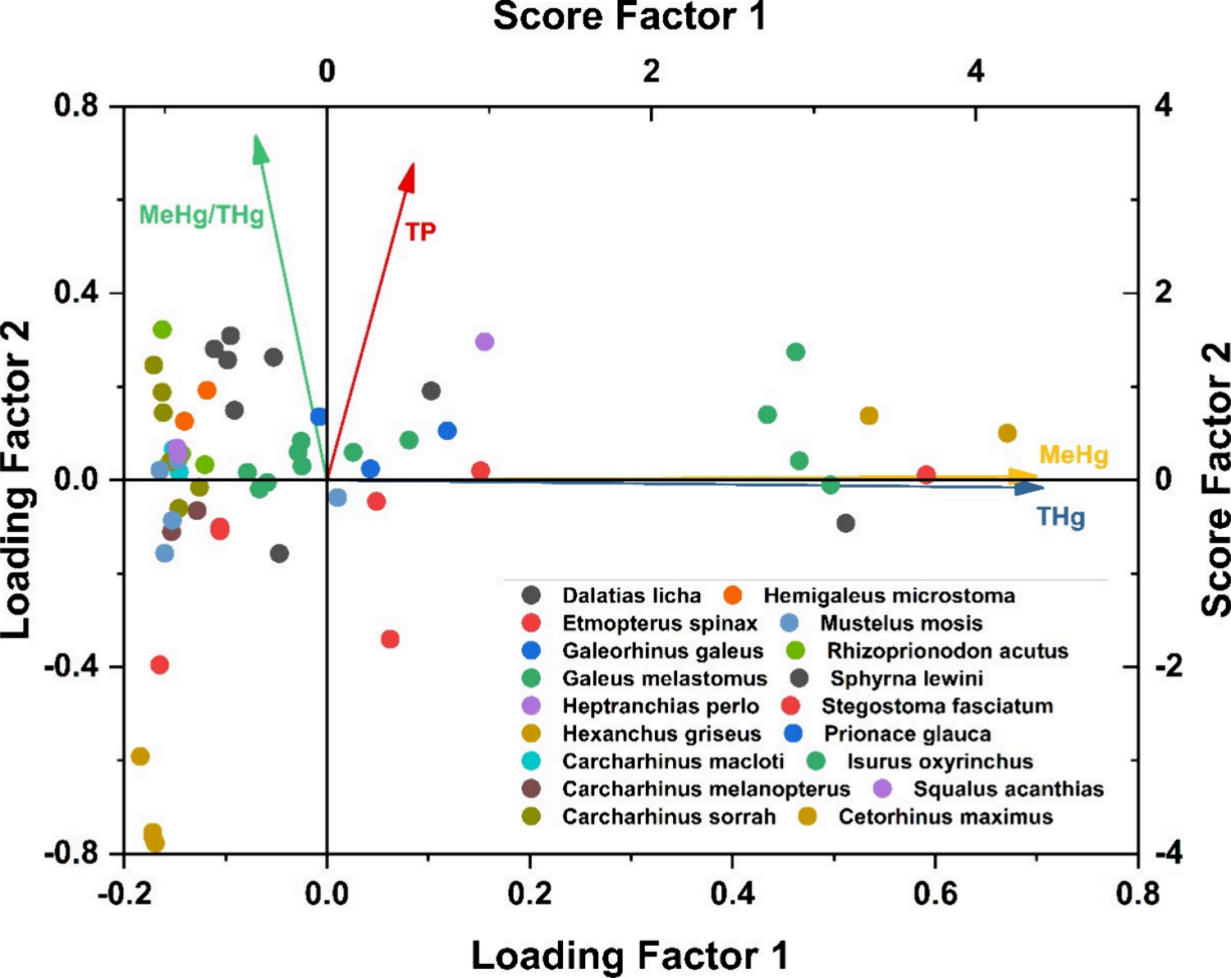
PCA biplot based on THg, MeHg, trophic position (TP), and MeHg/Hg in elasmobranch individuals (*n* = 63). PC1 (50% variance) reflects Hg bioaccumulation mainly driven by THg and MeHg loadings, while PC2 (37% variance) represents trophic-related gradients in %MeHg.

The high loading of both THg and MeHg on PC1 confirms that increases in total Hg are largely driven by MeHg bioaccumulation, consistent with the known preferential retention of MeHg in fish muscle.^44,45^ Individuals with the largest body size within each species clustered at high PC1 values: the largest *Galeus melastomus* (47–50 cm), *Etmopterus spinax* (42 cm) and *Sphyrna lewini* (217 cm), supporting strong size-dependent bioaccumulation over lifespan (the two individuals of *Hexanchus griseus* also cluster at high PC1 values, possibly reflecting species specific accumulation).

PC2 separated low-TP planktivorous species from high-TP predators, consistent with increasing MeHg proportion at higher trophic levels. No clear clustering by sex, habitat, or reproductive mode was observed, suggesting that Hg variability is primarily driven by bioaccumulation and trophic ecology.

THg ranged from 0.05–0.11 mg kg^–1^ in *C. macloti* to a maximum of 20.7 mg kg^–1^ observed in a single *H*. *griseus* individual. Low THg levels were found in *C. macloti*, *C. maximus* (0.07–0.11 mg kg^–1^) and *C. sorrah* (0.11–0.20 mg kg^–1^). Size-dependent accumulation was evident, as a larger *C. sorrah* (110 cm TL) had elevated THg (0.77 mg kg^–1^), in agreement with the PCA distribution along PC1.

Intermediate THg concentrations were found in *H. microstoma* (0.44–1.10 mg kg^–1^), *M. mosis* (0.26–4.60 mg kg^–1^), *R. acutus* (0.43–0.98 mg kg^–1^), and *C. melanopterus* (0.31–1.02 mg kg^–1^) (Supporting Information, Table S3). The limited variability observed in *R. acutus* and *C. melanopterus*, but also *C. maximus* and *C. sorrah* (except for the 110 cm TL individual), likely reflects the narrow size range and homogeneous life stages of the sampled individuals. For instance, *C. maximus* ranged from 390 to 437 cm TL, *C. melanopterus* from 59 to 61 cm TL, *R. acutus* from 78 to 85 cm TL, and *C. sorrah* from 68 to 90 cm TL.

Deep-sea predators, including *H. griseus*, *H. perlo*, *E. spinax*, and *G. melastomus*, exhibited substantially higher THg, consistent with their higher PC1 scores. However, while *E. spinax* and *G. melastomus* showed substantial variability in THg loads, associated with the wide size range of the sampled individuals (13–42 cm and 13.5–49.5 cm, respectively), *H. griseus* showed consistently high concentrations regardless of life stage (from neonates to adults).

Elevated THg concentration were also observed in large pelagic apex predators (*S. lewini* 1.5–17.4 mg kg^–1^, *P. glauca*: 3.7–5.1 mg kg^–1^ and *I. oxyrinchus:* 3.0–3.1 mg kg^–1^), indicative of biomagnification processes at higher trophic levels, but generally lower compared to their deep-sea counterparts (e.g., *H. griseus*) due to the reduced MeHg production and bioavailability in open-ocean environments.^17,46–48^ Juvenile *S. lewini* exhibited moderate THg (1.8–2.9 mg kg^–1^), whereas a single adult (>200 cm TL) reached 17.4 mg kg^–1^, comparable to *H. griseus* and prior reports (up to 18.9 mg kg^–1^ ^17^).

One-way ANOVA confirmed significant interspecific differences, with *E. spinax*, *S. lewini*, *M. mosis*, *G. melastomus*, and *H. microstoma* significantly higher than *C. maximus* (F­(7,44) = 21.632, *p* < 0.0001).

Habitat and diet are major drivers of THg variability in sharks.^17,23,37,47,49,50^ Deep-sea habitats act as a Hg sink, resulting in elevated concentrations in deep-sea megafauna.^48^ Mediterranean deep-sea sharks, including *H. griseus*, *H. perlo*, *G. melastomus*, *E. spinax*, and *D. licha*, exhibited the highest THg (Table S3), consistent with prior reports.^17,46,48^ Among this group, the highest THg levels were recorded in *H. griseus* and *H. perlo*, reflecting their diet primarily composed of other chondrichthyans, as well as teleost fish and marine mammals, including scavenging on whale carrion.^51,52^ Deleterious effects of THg exposure in deep-sea sharks, including impairing swimming efficiency and reproduction, have been reported even at lower concentrations,^53^ highlighting the need to further investigation of contamination in deep-sea assemblages.

Neritic species and filter-feeders (*C. macloti*, *C. sorrah*, *R. acutus*, *H. microstoma, C. maximus*) had lower THg concentrations, reflecting both habitats and diets (zooplankton, small teleosts, crustaceans, and cephalopods^54–56^ (Supporting Information, Table S1 and S3).

MeHg concentrations mirrored THg patterns (0.04 mg kg^–1^ in *C. maximus* to 17.7 mg kg^–1^ in *H. griseus*, Table S3), with a strong overall correlation (R^2^ = 0.99, *p* < 0.0001, Figure 2). Species-level correlations were significant in *C. sorrah* (R^2^ = 0.98, *p* < 0.0001), *E. spinax* (R^2^ = 0.98, *p* < 0.0001), *G. melastomus* (R^2^ = 0.98, *p* < 0.0001), *M. mosis* (R^2^ = 0.999, *p* < 0.0001), and *S. lewini* (R^2^ = 0.997, *p* < 0.0001). One-way ANOVA confirmed significant interspecific differences in MeHg concentrations, *E. spinax* and *G. melastomus* showing significantly higher levels than *C. maximus* (F­(7,44) = 22.551, *p* < 0.0001), consistent with THg analyses.

**Figure 2:**
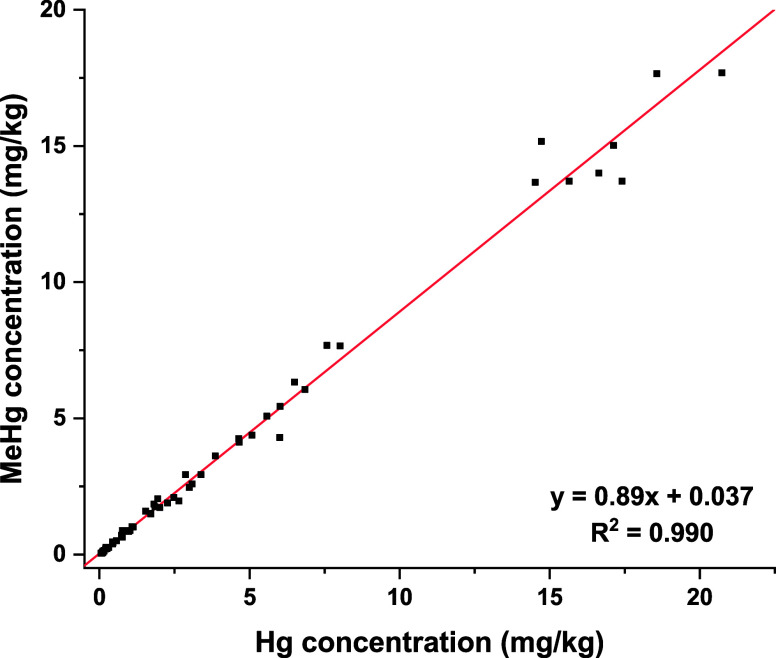
Robust linear regression of MeHg on THg (mg kg^–1^) in shark samples from the Mediterranean Sea, Indian and Atlantic Ocean.

MeHg-THg ratios ranged from 64.8% (*C. maximus*) to 101% (*H. perlo*), mean 89.7 ± 8.1% (Figure 3), confirming that MeHg dominates THg in shark muscle,^37,44,45,57^ but this substantial variation directly challenges the assumption of near-complete MeHg dominance across all shark taxa and highlights that diet and trophic ecology strongly influence interspecific variation.^28,47,58^ For instance, piscivorous sharks exhibited higher MeHg proportions than invertebrate feeders, with *Squalus mitsukurii* and *Galeorhinus australis* showing ∼90% MeHg compared to ∼75% in *Mustelus canis* and *Mustelus antarcticus*.^28,58^ A One-way ANOVA test followed by Tukey’s post hoc test revealed a significant interspecific difference in MeHg-THg ratios, with *C. maximus* having a statistically significant lower ratio than all other species (F­(7,44) = 4.699, p = 0.0008) (Figure 3). Expanding MeHg analyses across diverse shark species is essential to accurately characterize Hg speciation and avoid underestimating the species-specific toxicological risk of Hg in sharks.

**Figure 3:**
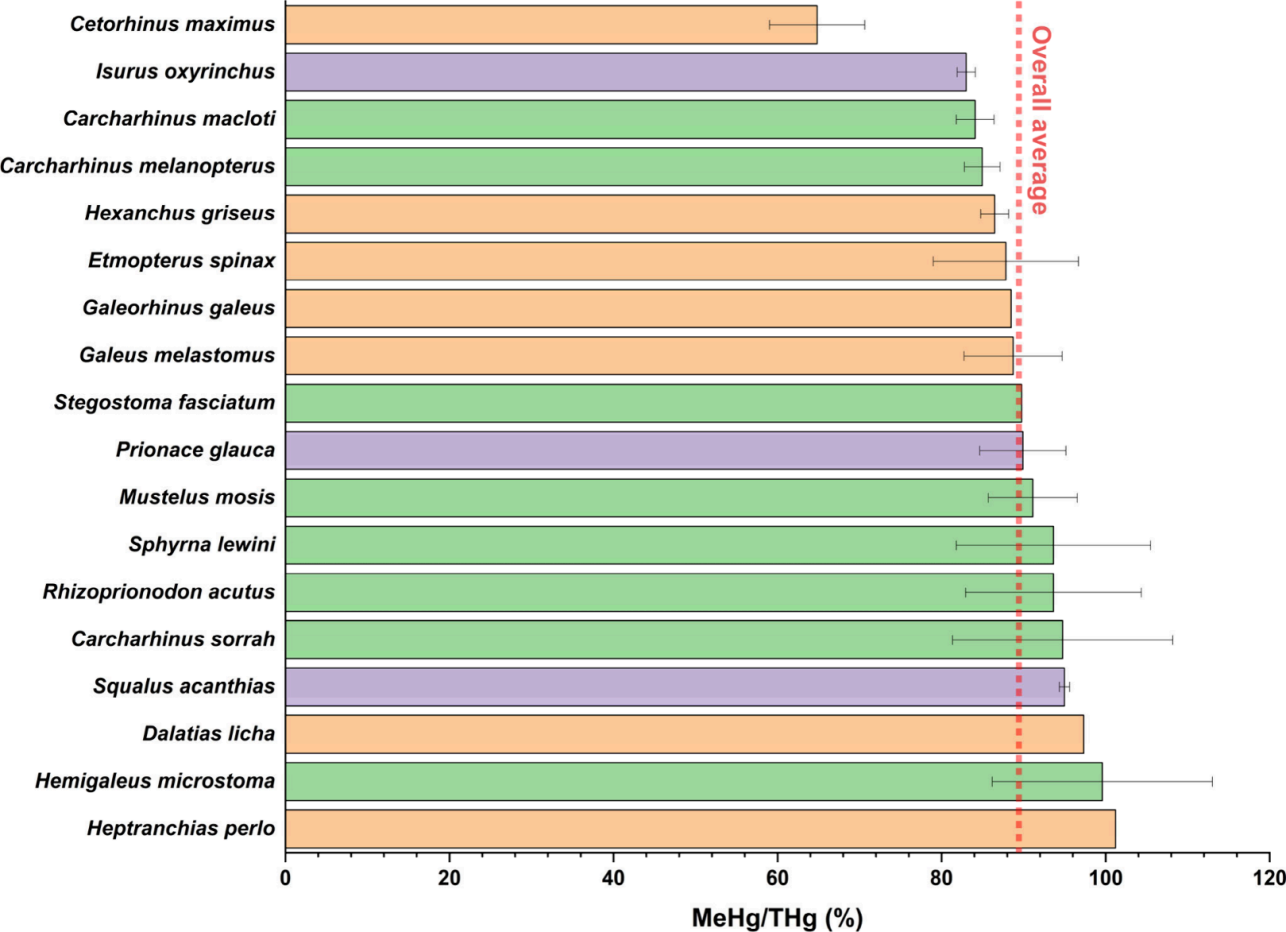
Comparison of MeHg-THg ratios among shark species, with colored bars indicating sampling regions: Mediterranean Sea (orange), Indian Ocean (green), and Atlantic Ocean (violet).

### 3.3 Biological Factors Influencing THg, MeHg Accumulation

Body size strongly influenced Hg accumulation, consistent with slow excretion and age-dependent bioaccumulation.^59,60^ Mercury binds strongly to sulfhydryl groups of proteins in marine fish, leading to a very slow excretion of Hg over time and a progressive increase in tissue concentrations with age and growth.^28^

Significant positive correlations were observed between size and THg/MeHg in *E. spinax* (R^2^ = 0.92, p = 0.003 and R^2^ = 0.88, p = 0.006, respectively), *G. melastomus* (R^2^ = 0.91, *p* < 0.001 and R^2^ = 0.92, *p* < 0.001, respectively), and *S. lewini* (R^2^ = 0.63, p = 0.031 and R^2^ = 0.68, p = 0.0235, respectively) (Figure 4). Bioaccumulation patterns were evident also considering individual body weight: *E. spinax* (THg: R^2^ = 0.98, *p* < 0.001 and MeHg: R^2^ = 0.98, *p* < 0.001) and *G. melastomus* (THg: R^2^ = 0.98, *p* < 0.001 and MeHg: R^2^ = 0.98, p = *p* < 0.001).

**Figure 4:**
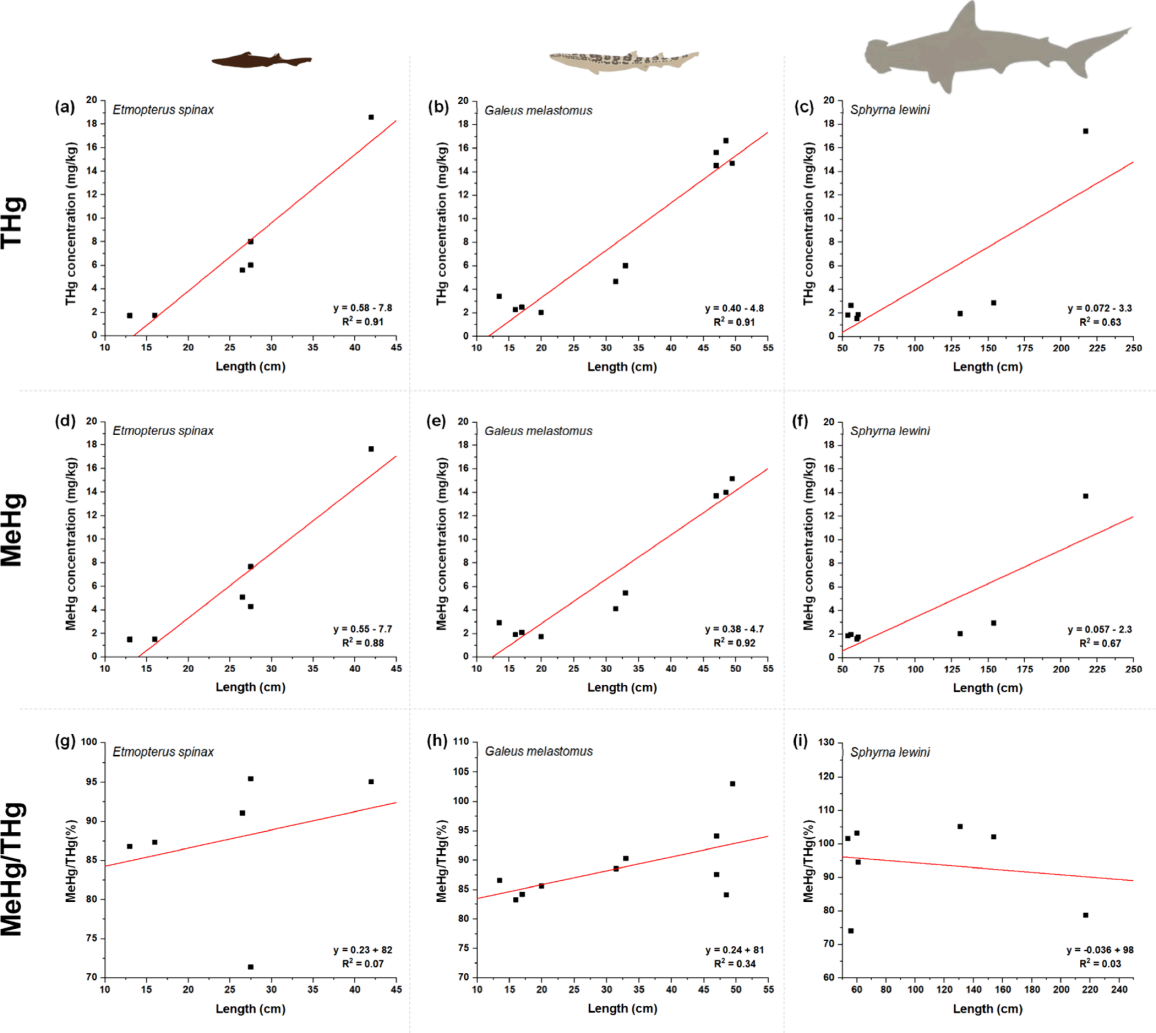
Correlation between THg, MeHg, and Mehg-THg ratio in *Etmopterus spinax* and *Galeus melastomus* from the Mediterranean Sea and *Sphyrna lewini* from the Indian Ocean.

The relationship between body size and Hg concentration is also influenced by ecological factors. Larger and older sharks often occupy higher trophic positions and exploit different habitats, depth ranges, and foraging grounds compared to smaller individuals.^54,61^ As sharks grow, they undergo ontogenetic dietary shifts, consuming prey at progressively higher trophic levels.^62–64^ For instance, in *E. spinax* juvenile individuals (13–16 cm) exhibited mean THg concentrations of approximately 1.7 mg kg^–1^, whereas larger specimens (42 cm) reached values up to 18.6 mg kg^–1^. This pattern is consistent with documented ontogenetic dietary shifts, whereby juveniles primarily consume small crustaceans and cephalopods, while adults increasingly prey on larger fish.^65^ A similar size-related trend was observed in *G. melastomus*. Individuals up to 30 cm, feeding mainly on cephalopods, showed THg concentrations of 2.5 ± 0.6 mg kg^–1^, which increased to 5.3 ± 1.0 mg kg^–1^ at intermediate sizes as prey size and trophic position increased. In larger specimens (>45 cm), which behave as generalist predators,^64,65^ THg concentrations reached 15.4 ± 1.0 mg kg^–1^. Therefore, the observed increase in THg with size likely results not only from bioaccumulation over time but also from ontogenetic shifts in diet and trophic position.^15^

To further explore Hg accumulation across trophic levels, TMF analysis in Mediterranean sharks indicated substantial biomagnification (THg: R^2^ = 0.87; MeHg: R^2^ = 0.88; *p* < 0.0001), with TMFs of 66.7 (THg) and 89.3 (MeHg). Excluding *C. maximus*, regressions remained significant (THg: R^2^ = 0.28, p = 0.0163; MeHg: R^2^ = 0.27, p = 0.035), confirming biomagnification and species-specific trophic effects consistent with PCA findings

Sex-specific effects were modest in agreement with previous findings,^15,28,66,67^ and were evaluated in the 8 species for which both sexes were available: females had higher THg and MeHg after size normalization (THg: t(10.49) = −2.53, p = 0.029; MeHg: t(11.29) = −2.32, p = 0.040), but no difference in MeHg/THg ratio. Consistently, the limited clustering by sex and reproductive strategy observed in the PCA suggests that these biological factors play a secondary role in shaping Hg variability compared with size, diet and trophic ecology.

Reproductive mode may also influence Hg accumulation in sharks. Previous studies have shown that, in viviparous species, contaminants can be transferred from the mother to developing embryos together with nutrients.^16^ In particular, placental viviparous sharks may facilitate continuous Hg transfer via placental nourishment, potentially contributing to partial maternal depuration.^16,28^ In contrast, this mechanism appears less effective or absent in species with alternative reproductive strategies, such as ovoviviparity. Consistent with this, no evidence of substantial maternal Hg transfer to eggs was reported in the ovoviviparous *Scyliorhinus canicula*, with egg Hg concentrations approximately 1 order of magnitude lower than those in maternal muscle.^68^ More recently, a recent study demonstrated higher Hg exposure in embryos of placental viviparous sharks compared to aplacental species, supporting the role of placentation as a key pathway for Hg transfer.^69^ Consistently, in this study yolk-sac placental viviparous species accumulated significantly lower THg and MeHg (Kruskal–Wallis test; THg: H = 9.02, d.f. = 2, n = 32, p = 0.011; MeHg: H = 9.26, d.f. = 2, n = 32, p = 0.0098), likely due to maternal transfer mechanisms.

### 3.4 Human Health Risk Assessment

While global shark fin trade has declined since the early 2000s, the trade in shark meat has grown steadily.^19^ At a global level, the European Union plays a front base role in the shark meat trade, with Spain as a major trader and Italy as a primary importer.^19^ Eight out of 18 species (44.4%) exceeded the EU maximum THg concentration (1 mg kg^–1^), a threshold adopted internationally and provide a conservative benchmark for global consumer risk (Figure 5).

**Figure 5:**
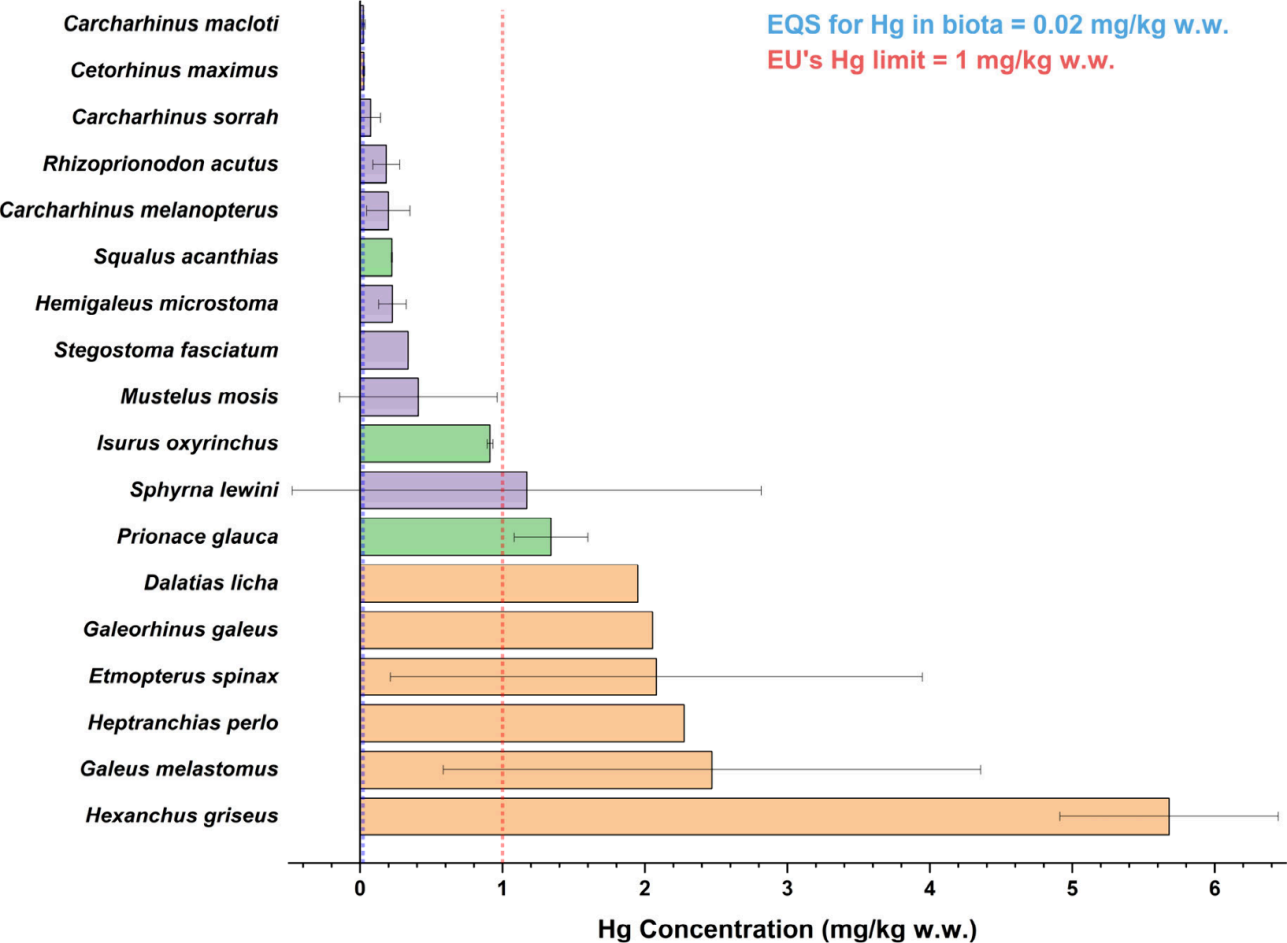
Concentrations of total mercury (THg) in shark species and comparison to international guidelines. Small dashed black lines: EU maximum concentration of THg in shark products (1 mg kg^–1^ ww); large dashed black lines: EQS_biota_ Hg limit (0.02 mg kg^–1^ ww).

Deep-sea and large pelagic apex predators, including *D. licha, E. spinax, G. melastomus, H. griseus, H. perlo, S. lewini, P. glauca*, and *I. oxyrinchus*, showed the highest risk. In particular, *D. licha* and *H. griseus* exceeded the limit even at the newborn stage, while *E. spinax* and *G. melastomus* only subadult and adult individuals. *S. lewini* also exceeded the limit; however, this was driven primarily by a single large adult female, as neonates and juveniles remained below the threshold. Neritic juveniles (e.g., *C. macloti*, *C. melanopterus* and *C. sorrah*) and filter-feeders remained below thresholds.

THQ analysis indicated that daily consumption would pose risks for 83.3% of species, while realistic weekly consumption led to THQ > 1 in 50% of species, primarily Mediterranean deep-sea sharks (Supporting Information, Table S4), indicating that regular consumption of deep benthic sharks could expose the human population to health risks.

While the THQs for most Indian Ocean species were < 1, *S. lewini* poses both a conservation concern, being Critically Endangered,^70^ and a potential health risk due to elevated Hg levels, and should therefore be avoided.^71,72^ For Atlantic Ocean species, THQs were >1 for both *P. glauca* and *I. oxyrinchus*, a finding of particular concern since fillets of these species were purchased directly from Italian food stores, highlighting the real potential for consumer exposure to elevated MeHg levels.

Half of analyzed species exceeded the Provisional Tolerable Weekly Intake for MeHg. These species included both deep-sea and pelagic apex predators, such as *D. licha*, *E. spinax*, *G. galeus*, *G. melastomus*, *H. perlo*, *H. griseus*, *S. lewini*, *P. glauca*, and *I.oxyrinchus*. Previous studies support this finding;^73^ for instance, Alves et al. reported that 32 out of 60 *Prionace glauca* sampled in Portugal exceeded the legal Hg limit, potentially exposing consumers to harmful MeHg amounts.

Maximum allowable monthly consumption limits (CR_mm_, assuming 16 meals per month) indicate that safe intake is highly restricted for many shark species, with nine species requiring <2 meals/month (Supporting Information, Table S5). A controlled consumption (2–10 meals month^–1^) would be required for most Indian Ocean species. Only *C. sorrah*, *C. macloti*, and *C. maximus* were considered low risk, demonstrating that risk management must consider species, size, and source region.

Finally, to have an indication of possible risks for the ecosystems as well, measured concentrations in sharks were checked against the Environmental Quality Standards EQS_biota_. All shark species exceeded EQS_biota_ thresholds (0.02 mg kg^–1^), indicating potential ecological risks through secondary poisoning in marine food webs (Figure 5).

Overall, our results show that the highest MeHg burdens occur in deep-sea and pelagic predators, species that are both heavily exploited and globally threatened, highlighting an urgent convergence of food-safety and conservation challenges.

### 3.5 Conservation Implications

Species with the highest THg concentrations (*G. galeus*, *I. oxyrinchus*, *S. lewini*) were also those classified as Endangered or Critically Endangered by the IUCN Red List, emphasizing dual conservation and public health challenge. Continued commercialization and consumption of such taxa not only undermine population recovery but also increase human exposure to MeHg. Aggravating the risk, shark meat remains widely marketed and consumed globally, often without consumers’ knowledge that they are purchasing or eating shark products, as documented in several Mediterranean countries.^74,75^ This unintentional consumption, driven largely by mislabeling and limited public awareness, represents a critical obstacle to effective risk mitigation. Therefore, alongside advising limited consumption, improving consumer awareness and improving labeling transparency is essential to reduce human exposure to MeHg and support more informed, sustainable seafood choices.^74^

## Supplementary Material



## Notes

Additional methodological details regarding the literature data collection and validation process. Table S1: Species traits (status, habitat, trophic level, and location). Table S2: 139 peer-reviewed sources (1975–2025) used for the meta-analysis. Figure S1: Global temporal distribution of these studies. Table S3: THg, MeHg, and %MeHg concentrations by species and reproductive mode. Tables S4 and S5: Human health risk metrics, including THQ, EWI, and EPA-based consumption limits (CRmm). Full bibliographic references for the data set. (PDF)
