# Supplementary material for: Global Patterns of Mercury Speciation and Biomagnification in Sharks: Ecological Drivers and Food Safety Implications
Source: Environ Sci Technol. 2026 May 8;60(20):14365–74. doi: 10.1021/acs.est.6c01257 (PMC13217547; doi:10.1021/acs.est.6c01257)
Supplement: Supplementary file 1 [file es6c01257_si_001.pdf]

Global Patterns of Mercury Speciation and Biomagnification in Sharks: Ecological Drivers and Food Safety Implications

Ginevra Boldrocchi[1,2], Davide Spanu[3], Alejandro Ruiz López[3], Fulvio Garibaldi[4], Luca Lanteri[4], Alberto Maggi[3], Roberta Bettinetti[1], and Damiano Monticelli[3*]*

[1] Department of Human Sciences, Innovation and Territory, University of Insubria, Via Valleggio 11, 22100 Como, Italy
[2] One Ocean Foundation, Via Gesù 10, 20121, Milan, Italy
[3] Department of Science and High Technology, University of Insubria, Via Valleggio 11, 22100 Como, Italy
[4] Department for Earth, Environment and Life Sciences, University of Genoa, Corso Europa 26, 16132 Genoa, Italy
*corresponding author: ginevra.boldrocchi@uninsubria.it; damiano.monticelli@uninsubria.it

Supporting Information

Additional Methodological Details

Literature Data Collection

Peer-reviewed studies reporting Hg or MeHg concentrations in elasmobranchs were included, without geographical or temporal restrictions. No geographical restrictions were applied on articles as long they were published in English and were considered studies on Hg and MeHg (Boldrocchi et al., 2023). Searches were conducted on Google Scholar and Scopus using the terms: "shark*", "elasmobranch*", "mercury*", "methylmercury", "heavy metal*", "MeHg*", "Hg*", "trace element", and "bioaccumulation", with Boolean operators (AND, OR, +). Reference lists were screened to capture additional studies, with literature up to September 2025 considered. For each study, we extracted authors, year, pollutants analyzed, taxa, study location, sampling date, MeHg:THg ratio, and tissue type. Muscle data were prioritized due to preferential Hg accumulation. Data were validated against Tiktak et al., (2020), with 93 overlapping studies enabling direct verification.

Table S1. List of collected species, status of conservation, habitat, trophic position, total length (cm) and the sample origin.

Family	Specie	IUCN (Global)	Habitat	TP	TL (range)	Fermale (%)	Location
Carcharhinidae	*Carcharhinus macloti*	NT	Neritic	4.2 ± 0.4	45-56	100	Indian Ocean
	Carcharhinus melanopterus	VU	Neritic	3.9 ± 0.4	59-61	50	Indian Ocean
	Carcharhinus sorrah	NT	Neritic	4.2 ± 0.5	68-100	60	Indian Ocean
	Prionace glauca	NT	Neritic/Oceanic	4.4 ± 0.2			Atlantic Ocean
	Rhizoprionodon acutus	VU	Neritic	4.3 ± 0.4	78-85	33	Indian Ocean
Cetorhinidae	*Cetorhinus maximus*	EN	Neritic/Oceanic	3.2 ± 0.3	390-437	50	Mediterranean Sea
Dalatiidae	*Dalatias licha*	VU	Oceanic/Deep Benthic	4.2 ± 0.4	32	100	Mediterranean Sea
Etmopteridae	*Etmopterus spinax*	VU	Oceanic/Deep Benthic	4.1 ± 0.2	13-42	83	Mediterranean Sea
Hemigaleidae	*Hemigaleus microstoma*	VU	Neritic	4.2 ± 0.4	82-94	67	Indian Ocean
Hexanchidae	*Heptranchias perlo*	NT	Neritic/Oceanic/Deep Benthic	4.2 ± 0.4			Mediterranean Sea
	Hexanchus griseus	NT	Deep Benthic	4.5 ± 0.2	65-400	100	Mediterranean Sea
Lamnidae	*Isurus oxyrinchus*	EN	Oceanic	4.5			Atlantic Ocean
Pentanchidae	*Galeus melastomus*	LC	Neritic/Deep Benthic	4.2 ± 0.3	13-5-49.5	50	Mediterranean Sea
Sphyrnidae	*Sphyrna lewini*	CR	Neritic/Oceanic	4.1 ± 0.5	54-217	50	Indian Ocean
Squalidae	*Squalus acanthias*	VU	Neritic/Oceanic/Deep Benthic	4.4 ± 0.4			Atlantic Ocean
Stegostomidae	*Stegostoma fasciatum*	EN	Neritic/Oceanic	3.1 ± 0.4	170	100	Indian Ocean
Triakidae	*Galeorhinus galeus*	CR	Neritic/Oceanic/ Deep Benthic	4.3 ± 0.1		100	Mediterranean Sea
	Mustelus mosis	NT	Neritic/Deep Benthic	4.0 ± 0.52	24-38	100	Indian Ocean

Table S2. List of peer-reviewed articles published on mercury and methylmercury levels in shark muscles from 1975 to 2025 (N = 139)

Article	Authors	Sampling Date	Location	Species	MgHg	Hg
1	Adams and Mc Michael, 1999	1992-1995	Florida, USA, Atlantic Ocean	*Carcharhinus leucas*		x
				Carcharhinus limbatus		x
				Rhizoprionodon terraenovae		x
				Sphyrna tiburo		x
2	Adel et al., 2016	2014	Persian Gulf, Arabian Sea	*Carcharhinus dussumieri*		x
3	Adel et al., 2017	2014-2015	Persian Gulf, Arabian Sea	*Rhizoprionodon acutus*		x
4	Adel et al., 2018	2015-2016	Persian Gulf, Arabian Sea	*Chiloscyllium arabicum*		x
5	Al Ali et al., 2025	2020	Arabian Gulf, Arabian Sea	*Rhizoprionodon acutus*		x
				Carcharhinus sorrah		x
6	Al-Reasi et al., 2007	2004	Gulf of Oman, Arabian Sea	*Rhizoprionodon acutus*	x	x
7	Alves et al., 2016	-	Portugal, Atlantic Ocean	*Prionace glauca*		x
8	Amorim-Lopes et al., 2020	2017-2019	Brazil, Atlantic Ocean	*Rhizoprionodon lalandii*		x
				Rhizoprionodon porosus		x
9	Baeyens et al., 2003	-	North Sea	*Scyliorhinus canicula*	x	x
10	Barò-Camarasa et al., 2022	2017-2018	Baja California, Mexico, Pacific Ocean	*Rhizoprionodon longurio*		x
				Mustelus henlei		x

11	Bendall et al., 2014	2011	Celtic Sea	*Lamna nasus*		x
12	Bergés-Tiznado et al., 2015	2011	Gulf of California, Pacific Ocean	*Sphyrna lewini*		x
13	Besnard et al., 2021	2014-2016	Baja California, Mexico, Pacific Ocean	*Prionace glauca*		x
				Sphyrna zygaena		x
				Isurus oxyrinchus		x
14	Besnard et al., 2023	2009 and 2014-2018	Baja California, Mexico, Pacific Ocean	*Sphyrna zygaena*		x
15	Biton-Posmoguer et al., 2018	2012-2013	Spain and Portugal, Atlantic Ocean	*Prionace glauca*		x
				Isurus oxyrinchus		x
16	Biton-Porsmoguer et al., 2024	2010	Iceland, Greenland Sea	*Somniosus microcephalus*		x
17	Blanco et al., 2008	-	Spain (commercial samples)	*Isurus oxyrinchus*		x
				Prionace glauca		x
18	Boldrocchi et al., 2019	2016-2017	Djibouti, Indian Ocean	*Rhizoprionodon acutus*		x
				Sphyrna lewini		x
19	Boldrocchi et al., 2021	2016-2019	Djibouti, Indian Ocean	*Carcharhinus falciformis*		x
				Carcharhinus sorrah		x
				Carcharhinus limbatus		x
				Carcharhinus melanopterus		x
				Carcharhinus macloti		x
				Rhizoprionodon acutus		x
				Hemigaleus microstoma		x
				Hemipristis elongatus		x
				Sphyrna lewini		x
				Mustelus mosis		x
				Stegostoma fasciatum		x
20	Boldrocchi et al., 2022	2006-2020	Italy, Mediterranean Sea	*Cetorhinus maximus*		x
21	Bosch et al., 2016	-	South Africa, Atlantic Ocean	*Mustelus mustelus*	x	x
22	Branco et al., 2004	-	Azores Islands, Portugal, Atlantic Ocean	*Prionace glauca*	x	x
			Canarias, Spain, Atlantic Ocean	*Prionace glauca*	x	x
23	Branco et al., 2007	2004-2005	Azores Island, Portugal, Atlantic Ocean	*Prionace glauca*	x	x
24	Burger et al., 2011	2005-2006	New Jersey, USA, Atlantic Ocean	*Isurus oxyrinchus*		x
25	Cagnazzi et al., 2019	-	Australia, Pacific Ocean	*Sphyrna mokarran*		x
				Carcharhinus obscurus		x
				Carcharias taurus		x
				Carcharodon carcharias		x
				Carcharhinus leucas		x
				Mobula kuhlii		x
				Aetobatus ocellatus		x
				Rhynchobatus australiae		x
				Rhinoptera neglecta		x
26	Cai et al., 2007	2002-2003	Gulf of Mexico, Pacific Ocean	*Carcharhinus spp.*		x
27	Campos et al., 2024	2015, 2018, 2022 and 2023	Brazil, Atlantic Ocean	*Carcharhinus sp.*	x	x
				C. acronotus	x	x
				C. limbatus	x	x
				Galeocerdo cuvier	x	x
				Ginglymostoma cirratum	x	x
				Mustelus canis	x	x
				Heptranchias perlo	x	x

				Rhizoprionodon lalandii	x	x
				R. porosus	x	x
28	Chouvelon et al., 2012	2001-2010	France, Atlantic Ocean	*Scyliorhinus canicula*		x
				Galeus melastomus		x
				Mustelus asterias		x
				Mustelus mustelus		x
				Etmopterus spinax		x
				Centroselachus crepidater		x
				Deania calcea		x
				Deania profundorum		x
29	Chouvelon et al., 2018	2008 and 2012	France, Mediterranean Sea and Atlantic Ocean	*Scyliorhinus canicula*	x	x
				Galeus melastomus	x	x
30	Coehlo et al., 2010	2007	Atlantic Ocean	*Scyliorhinus canicula*	x	x
31	Corsolini et al., 2014	2010	Greenland, Atlantic Ocean	*Somniosus microcephalus*		x
32	Crawford et al., 2023	2018-2021	Canada, Atlantic Ocean	*Carcharodon carcharias*		x
33	Cresson et al., 2014	2012	Gulf of Lions, France, Mediterranean Sea	*Galeus melastomus*		x
				Scyliorhinus canicula		x
34	Davis et al., 2016	2009-2010	California, USA, Pacific Ocean	*Mustelus henlei*		x
				Triakis semifasciata		x
35	de Alencar Goyanna et al., 2025	2020-2022	Brazil, Atlantic Ocean	*Isurus oxyrinchus*		x
				Prionace glauca	x	x
36	de Carvalho et al., 2014	2006-2007	Brazil, Atlantic Ocean	*Prionace glauca*	x	x
37	De Loose et al., 2025	2024	Albania, Mediterranean Sea	*Mustelus punctulatus*		x
38	De Moura et al., 2015	2009	Brazil, Atlantic Ocean	*Megachasma pelagios*	x	x
39	de Pinho et al., 2002	1997	Brazil, Atlantic Ocean	*Carcharhinus signatus*		x
				Mustelus canis	x	x
				Mustelus norrisi		x
				Squalus megalops		x
				Squalus mitsukuri	x	x
40	de Pinho et al., 2025	2021-2022	Brazil, Atlantic Ocean	*Isurus oxyrinchus*		x
41	de Souza Picaluga et al., 2025	2015-2017	Brazil, Atlantic Ocean	*Prionace glauca*	x	x
42	Dutton et al., 2019	2013	New York, USA, Atlantic Ocean	*Alopias vulpinus*		x
43	Dutton et al., 2023	1995	Taiwan, Pacific Ocean	*Whale Shark*		x
44	Ehnert-Russo and Gelsleichter, 2019	2014-2017	USA, Atlantic Ocean	*Rhizoprionodon terraenovae*		x
45	Elsayed et al., 2020	2017	Arabian Gulf, Arabian Sea	*Rhizoprionodon oligolinx*	x	
				Chiloscyllium arabicum	x	
46	Endo et al., 2008	2007	Japan, Pacific Ocean	*Galeocerdo cuvier*		x
47	Endo et al., 2009	2008	Japan, Pacific Ocean	*Squalus acanthias*		x
48	Endo et al., 2013	2008	Japan, Pacific Ocean	*Mustelus manazo*		x
49	Endo et al., 2016	2007-2014	Japan, Pacific Ocean	*Carcharhinus albimarginatus*		x
50	Escobar-Sanchez et al., 2010	2006-2007	Baja California Sur, Mexico, Pacific Ocean	*Sphyrna zygaena*		x
51	Escobar-Sánchez et al., 2011	2006-2007	Baja California Sur, Mexico, Pacific Ocean	*Prionace glauca*		x
52	Ferreira et al., 2004	1995-1999	Brazil, Atlantic Ocean	*Carcharhinus signatus*		x
53	Forsyth et al., 2004	-	Canada (commercial samples)	*Carcharhinus limbatus*	x	x
				Undefined Species	x	x
54	Frías-Espericueta et al., 2015	2012	Gulf of California, Mexico, Pacific Ocean	*Rhizoprionodon longurio*		x
55	Frías-Espericueta et al., 2019	-	Gulf of California, Mexico, Pacific Ocean	*Rhizoprionodon longurio*		x

56	Gaion et al., 2016	2012	Italy, Mediterranean Sea	*Galeus melastomus*		x
57	Gallo et al., 2025	2010-2021	Italy, Mediterranean Sea	*C. granulosus*		x
				Dalatias licha		
				Galeorhinus galeus		
				Galeus melastomus		
				Hexanchus griseus		
				Heptranchias perlo		
				Isurus oxyrinchus		
				Prionace glauca		
				Squalus acanthias		
				Scyliorhinus canicula		
58	García-Hernández et al., 2007	2003-2004	Gulf of California, Mexico, Pacific Ocean	*Sphyrna zygaena*		x
				Alopias pelagicus		x
				Rhizoprionodon longurio		x
				Carcharhinus obscurus		x
				Sphyrna lewini		x
				Carcharhinus limbatus		x
				Carcharhinus falciformis		x
				Prionace glauca		x
				Mustelus henlei		x
				Triakis semifasciata		x
				Nasolamia velox		x
59	Gelsleichter et al., 2020	2017	Bahamas, Atlantic Ocean	*Carcharhinus longimanus*		x
60	Gholamhosseini et al., 2025	2023-2024	Persian Gulf, Iran, Arabian Sea	*Rhizoprionodon acutus*		x
				Rhizoprionodon oligolinx		x
				C. dussumieri		x
				C. arabicum		x
61	Gilbert et al., 2015	2013	Australia, Tasman Sea, Pacific Ocean	*Carcharhinus obscurus*		x
				C. plumbeus		x
				Carcharodon carcharias		x
62	Giovos et al., 2025	2023	Greece, Mediterranean Sea	*Mustelus mustelus*		x
63	Greenfield et al., 2005	1997-2000	San Francisco, USA, Pacific Ocean	*Triakis semifasciata*		x
64	Greig et al., 1975	1971	Massachusetts, USA, Altantic Ocean	*Squalus acanthias*		x
65	Hammerschlag et al., 2016	-	Atlantic and Pacific Oceans	*Carcharhinus acronotus*	x	x
				Carcharhinus limbatus	x	x
				Sphyrna tiburo	x	x
				Carcharhinus leucas	x	x
				Sphyrna mokarran		x
				Negaprion brevirostris	x	x
				Ginglymostoma cirratum	x	x
				Rhizoprionodon terraenovae	x	x
				Sphyrna zygaena		x
				Galeocerdo cuvier		x
66	Higueruelo et al., 2025	2019-2023	Spain, Mediterranean Sea	*Galeus melastomus*		x
				S. canicula		x
67	Hornung et al., 1993	1985-1991	Israel, Mediterranean Sea	*Centrophorus granulosus*		x
				Galeus melastomus		x
				Sornniosus rostratus		x

				Etmopterus spinax		x
				Hexanchus griseus		x
68	Hueter et al., 1995	1988-1992	Florida, USA, Atlantic Ocean	*Carcharhinus acronotus*	x	
				Carcharhinus limbatus	x	
				Carcharhinus leucas	x	
				Carcharhinus perezi	x	
				Carcharhinus obscurus	x	
				Carcharhinus plumbeus	x	
				Carcharhinus falciformis	x	
				Carcharhinus brevipinna	x	
				Galeocerdo cuvier	x	
69	Hurtado-Banda et al., 2012	2009-2010	Mexico, Pacific Ocean	*Sphyrna lewini*		x
				Rhizoprionodon longurio		x
70	Julio et al., 2022	2014-2018	Brazil, Atlantic Ocean	*Rhizoprionodon porosus*		x
71	Kaneko and Ralston., 2007	2006	Hawaii, USA, Pacific Ocean	*Alopius vulpinus*		x
				Isurus oxyrinchus		
72	Karimi et al., 2013	2007-2008	New York, USA, Altantic Ocean	*Alopias vulpinus*		x
				Isurus oxyrinchus		x
73	Kim et al., 2019	2017	South Korea, Pacific Ocean	*Carcharhinus brachyurus*	x	x
				Carcharhinus obscurus	x	x
				Isurus oxyrinchus	x	x
				Triakis scyllium	x	x
				Mustelus manazo	x	x
				Cephaloscyllium umbratile	x	x
74	Kiszka et al., 2015	2009-2010	From Madagascar to Mascarene Islands, Indian Ocean	*Prionace glauca*		x
				Isurus oxyrinchus		x
				Carcharhinus longimanus		x
				Sphyrna lewini		x
				Alopias pelagicus		x
				Pseudocarcharias kamoharai		x
				Carcharhinus falciformis		x
75	Kousteni et al., 2006	2003-2004	Greece, Mediterranean Sea	*Squalus acanthias*		x
				Mustelus mustelus		x
76	Lacerda et al., 2000	-	Brazil, Atlantic Ocean	*Rhizoprionodon lalandei*		x
				R. porosus		x
				Mustelus higmani		x
77	Le Croizier et al., 2020	2016-2018	Guadalupe Island, Mexico, Pacific Island	*Carcharodon carcharias*		x
78	Li et al., 2022	2019-2020	Tropical Pacific Ocean	*Carcharhinus falciformis*		x
79	Li et al., 2023	2019-2020	Pacific Ocean	*Alopias superciliosus*	x	x
				Alopias pelagicus	x	x
80	Liu et al., 2023	2020-2021	Keelung, Taipei and Penghu, Pacific Ocean	*Prionace glauca*	x	
				Isurus oxyrinchus	x	
				Sphyrna zygaena	x	
				Alopias superciliosus	x	
				Rhynchobatus australiae	x	
				Carcharhinus limbatus	x	
				Hemitriakis japanica	x	
81	Lloret-Lloret et al., 2025	2019	Spain, Mediterranean Sea	*Scyliorhinus canicula*		x

82	Lopez et al., 2013	2011	Chile, Pacific Ocean	*Prionace glauca*		x
				Isurus oxyrinchus		x
83	Lyons and Lowe 2013	2012	California, USA, Pacific Ocean	*Alopias vulpinus*		x
84	Lyons et al., 2013	2006-2012	South California, USA, Pacific Ocean	*Carcharodon carcharias*		x
				Isurus oxyrinchus		x
				Lamna ditropis		x
				Alopias vulpinus		x
85	Marsico et al., 2007	-	Brazil, Atlantic Ocean	*Prionace glauca*		x
				Isurus oxyrhinchus		x
				Sphyrna zygaena		x
86	Matos et al., 2015	-	Portugal, Atlantic Ocean	*Prionace glauca*	x	x
87	Matulik et al., 2017	2009-2010	Florida, USA, Atlantic Ocean	*Carcharhinus acronotus*	x	x
				C. limbatus	x	x
				Negaprion brevirostris	x	x
				C. leucas	x	x
88	Maurice et al., 2021	2017	Galapagos Islands, Ecuador, Pacific Ocean	*Sphyrna lewini*		x
				Prionace glauca		x
				Carcharhinus longimanus		x
				C. falciformis		x
				Alopias superciliosus		x
				A. pelagicus		x
89	Maynard and Baumann, 2020	2018	Massachusetts, USA, Atlantic Ocean	*Squalus acanthias*	x	x
90	Maz-Courrau et al., 2012	2001-2005	Baja California Sur, Mexico, Pacific Ocean	*Carcharhinus falciformis*		x
				Prionace glauca		x
				Sphyrna zygaena		x
				Isurus oxyrinchus		x
91	McKinney et al., 2016	2005-2010	South Africa, Indian Ocean	*Carcharhinus amboinensis*		x
				Carcharhinus brachyurus		x
				Carcharhinus brevipinna		x
				Carcharhinus limbatus		x
				Carcharhinus obscurus		x
				Carcharhinus plumbeus		x
				Galeocerdo cuvier		x
				Rhizoprionodon acutus		x
				Sphyrna lewini		x
				Sphyrna zygaena		x
				Mustelus mosis		x
				Alopias vulpinus		x
				Carcharodon carcharias		x
				Isurus oxyrinchus		x
				Carcharias taurus		x
				Rhincodon typus		x
92	McMeans et al., 2015	2007-2009	Cumberland Sound, Nunavut, Canada, Arctic and Atlantic Oceans	*Somniosus microcephalus*		x
93	Medina-Morales et al., 2020	2015-2017	Mexico, Pacific Ocean	*Mustelus henlei*		x
94	Mohammed and Mohammed, 2017	-	Trinidad and Tobago, Atlantic Ocean	*Sphyrna lewini*		x
				Caraharinus porosus		x
95	Mol et al., 2001	1997-2000	Suriname, Atlantic Ocean	*Carcharhinus acronotus*		x
				Carcharhinus limbatus		x

				Mustelus canis		x
				Mustelus higmani		x
96	Moore et al., 2015	2011	Arabian Gulf, Kuwait, Arabian Sea	*Carcharhinus leiodon*		x
97	Mull et al., 2012	-	California, USA, Pacific Ocean	*Carcharodon carcharias*		x
98	Nam et al., 2011	2007-2008	Florida,USA, Atlantic Ocean	*Negaprion brevirostris*		x
99	Nicolaus et al., 2016	2014	Celtic Sea, Atlantic Ocean	*Lamna nasus*		x
100	Núñez-Nogueira, 2005	1994-1995	Veracruz, Mexico, Atlantic Ocean	*Carcharhinus limbatus*		x
				Rhizoprionodon terraenovae		x
101	O'Bryhim et al., 2017	2012-2013	St. Catherine's Island, Georgia, USA, Atlantic Ocean	*Sphyrna tiburo*		X
				Carcharhinus falciformis		X
102	Ouédraogo and Amyot, 2011	2010	Montreal, Canada (Commercial Samples)	*Scyliorhinus canicula*		x
103	Paiva et al., 2012	2008-2009	Portugal, Atlantic Ocean	*Deania calcea*		x
104	Pantoja-Echevarría et al., 2021	2018	Baja California Sur, Mexico, Pacific Ocean	*Mustelus henlei*		x
105	Pantoja-Echevarría et al., 2023	2018	Baja California Sur, Mexico, Pacific Ocean	*Mustelus henlei*		x
106	Pantoja-Echevarría et al., 2024	2018	Baja California Sur, Mexico, Pacific Ocean	*Mustelus californicus*		x
				Sphyrna zygaena		x
				I. oxyrinchus		x
				Mustelus henlei		x
107	Pethybridge et al., 2010	2004-2006	Tasmania, South of Victoria and South Australia, Australia, Pacific and Indic Ocean	*Etmopterus baxteri*	x	x
				Centroselachus crepidater	x	x
				Centroscymnus owstoni	x	x
				Centroscymnus coelopsis		x
				Deania calcea		x
				Dalatias licha	x	x
				Proscymnodon plunketi	x	x
				Apristurus sp		x
				Centrophorus zeehaani		x
				Squalus acanthias	x	x
				Squalus megalops	x	x
				Squalus mitsukurii	x	x
				Figaro boardmani		x
				Notorynchus cepedianus		x
108	Pethybridge et al., 2012	2004-2006	Tasmania and South of Victoria, Australia, Pacific and Indic Ocean	*Squalus acanthias*		x
				Squalus megalops		x
				Notorynchus cepedianus		x
				Centrophorus zeehaani		x
				Squalus mitsukurii		x
				Figaro boardmani		x
				Apristurus sinensis		x
				Centroselachus crepidater		x
				Centroscymnus owstoni		x
				Centroscymnus coelopsis		x
				Deania calcea		x
				Dalatias licha		x
				Etmopterus baxteri		x
				Proscymnodon plunketi		x
109	Powell and Powell, 2000	1977–1987	Papua New Guinea, Pacific Ocean	*Carcharhinus limbatus*		x

				Rhizoprionodon acutus		x
				Sphyrna lewini		x
110	Rechimont et al., 2024	2019	Gulf of California, Mexico, Pacific Ocean	Prionace glauca		x
111	Rechimont et al., 2025	2019-2021	Mexico, Pacific Ocean	Prionace glauca		x
112	Reinero et al., 2025	2024	Greenland, North Atlantic Ocean	Somniosus microcephalus		x
113	Reistad et al., 2021	2016	Florida, USA, Atlantic Ocean	Carcharhinus limbatus		x
114	Riesgo et al., 2023	2017-2018	Canary Islands, Spain, Atlantic Ocean South of Portugal, Atlantic Ocean Spain, Mediterranean Sea	Prionace glauca		x
115	Rodrigues et al., 2022	2016	Portugal, Atlantic Ocean	Etmopterus spinax		x
116	Rodríguez-Gutiérrez et al., 2020	2014-2015	Chiapas, Mexico, Pacific Ocean	Carcharhinus falciformis		x
117	Roubie et al., 2024	2015-2016	Aegan, Ionian and Lybian Seas, Mediterranean Sea	Mobula mobular		x
				Oxynotus centrina		x
				Heptranchias perlo		x
				Hexanchus griseus		x
				Hexanchus nakamurai		x
				Alopias superciliosus		x
				Prionace glauca		x
				Sphyrna zygaena		x
				Odontaspis ferox		x
				Isurus oxyrinchus		x
118	Rumbold et al., 2014	2010-2013	Florida, USA, Atlantic Ocean	C. acronotus		x
				C. limbatus		x
				C. leucas		x
				S. mokarran		x
				N. brevirostris		x
				Rhizoprionodon terraenovae		x
				Galeocerdo cuvier		x
119	Sadhu et al., 2015	-	Dunedin, New Zealand, Pacific Ocean	S. acanthias	x	x
				Galeorhinus galeus	x	x
120	Sandoval-Herrera et al., 2016	2010-2011	Costa Rica, Pacific Ocean	Mustelus henlei		x
				Scyliorhinus canicula		x
121	Sisma-Ventura et al., 2024	1985–2022	Israel, Mediterranean Sea	Centrophorus granulosus		
				Etmopterus spinax		
				Dalatias licha		
				Somniosus rostratus		
122	Storelli et al., 2002	1999	Italy, Mediterranean Sea	Galeus melastomus	x	x
				Scyliorhinus canicula	x	x
				Dalatias licha	x	x
				Centrophorus granulosus	x	x
				Sqaulus balinvillei	x	x
				Etmopterus spinax	x	x
				Mustelus mustelus	x	x
				Heptranchias perlo	x	x
				Sphyrna zygaena	x	x
123	Storelli at al., 2003	2001	Ionian Sea, Mediterranean Sea	Sphyrna zygaena	x	x
124	Storelli et al., 2005	2003	Italy, Adriatic Sea, Mediterranean Sea	Scyliorhinus canicula	x	x
125	Storelli et al., 2011	2009	Italy, Adriatic Sea, Mediterranean Sea	Mustelus mustelus		x

126	Storelli et al., 2022	2020	Italy, Mediterranean Sea	*Prionace glauca*	x	x
				Squalus acanthias	x	x
				Squalus blainville	x	x
				Mustelus mustelus	x	x
				Mustelus asterias	x	x
				Scyliorhinus canicula	x	x
				Lamna nasus	x	x
127	Suk et al., 2009	1991-1992<br>2004-2005	Hawaii, USA, Pacific Ocean<br>California, USA, Pacific Ocean	*Alopias vulpinus*		x
				Isurus oxyrinchus		x
128	Taylor et al., 2014	2009-2012	New England, USA, Atlantic Ocean	*Mustelus canis*		x
				Squalus acanthias		x
				Leucoraja erinacea		x
129	Teffer et al., 2014	2008-2011	New England, USA, Atlantic Ocean	*Isurus oxyrinchus*		x
				Alopias vulpinus		x
130	Terrazas-Lopez et al., 2019	2014	Baja California Sur, Mexico, Pacific Ocean	*Carcharhinus falciformis*		x
				Sphyrna zygaena		x
131	Torres et al., 2014	2013	Azores Islands, Portugal, Atlantic Ocean	*Galeorhinus galeus*		x
132	Torres-Escribano et al., 2011	2010	Valencia, Spain, Mediterranean Sea	*Galeorhinus galeus*		x
133	Velez et al., 2021	2009-2013	Colombia, Pacific Ocean	*Alopias pelagicus*		x
				Sphyrna lewini		x
				Carcharhinus cerdale		x
				Sphyrna corona		x
				Mustelus lunulatus		x
				Mustelus henlei		x
				Sphyrna tiburo		x
134	Vélez-Alavez et al., 2013	2008	Baja California Sur, Mexico, Pacific Ocean	*Isurus oxyrinchus*		x
135	Walker, 1988	1972-1978	Australia, Southern and Pacific Oceans	*Isurus oxyrinchu*		x
				Centrophorus uyato		x
				Squalus blainvillei		x
				Heptranchias perlo		x
				Squalus megalops		x
				Carcharhinus brevipinna		x
				Notorynchus cepedianus		x
				Carcharodon carcharias		x
				Cephaloscyllium laticeps		x
				Sphryna zygaena		x
				Squalus acanthias		x
				Furgaleus macki		x
				Galeorhinus galeus		x
				Asymbolus vincenti		x
				Carcharhinus brachyurus		x
				Parascyllium ferrugineum		x
				Pristiophorus cirratus		x
				Pristiophorus nudipinnus		x
				Mustelus antarcticus		x
				Heterodontus portusjacksoni		x
				Prionace glauca		x
				Parascyllium variolatum		x

				Alopim vulpinus	x
				Squatina australis	x
136	Walker et al., 2014	1998-2001	Florida, USA, Atlantic Ocean	*Sphyrna tiburo*	x
137	Wang et al., 2023	2015-2018	Indian Ocean	*Carcharhinus falciformis*	x
138	Wosnick et al., 2021	2018-2019	Brazil, Atlantic Ocean	*Ginglymostoma cirratum*	x
139	Zaera and Johnsen, 2011	2006	Angola, Atlantic Ocean	*Mustelus mustelus*	x

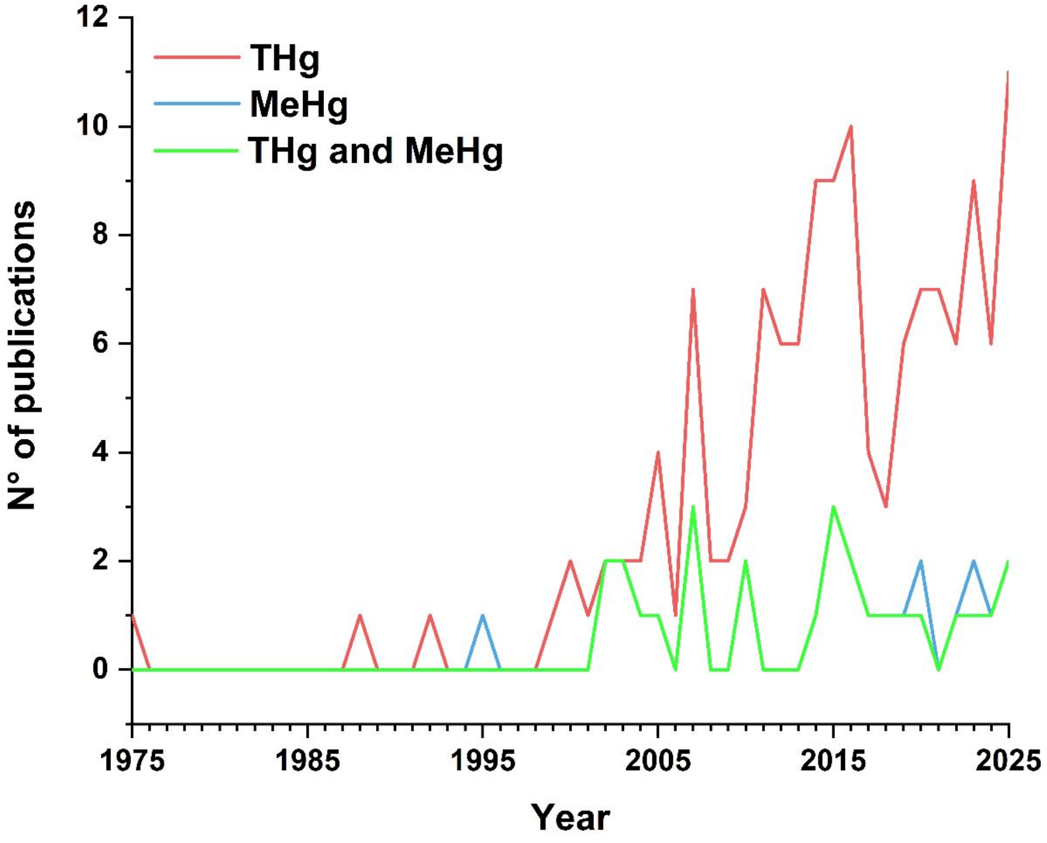

Figure S1. Temporal distribution of publications focusing on total mercury THg (red line), methylmercury MeHg (green line) and THg and MeHg (blue line) in shark muscles globally from 1975 to 2025 (N = 139).

Table S3. Total mercury (THg), methylmercury (MeHg), and MeHg-THg ratio in muscles of shark species. Data are means±sd expressed on a dry weight basis (mg kg^{-1}).

	Species	N	THg	MeHg as Hg	% Ratio	Reproductive mode
**1**	*Dalatias licha*	1	6.5	6.3	97.3	Aplacental viviparous
**2**	*Etmopterus spinax*	6	6.9 ± 6.2	6.3 ± 6	87.8 ± 8.9	Aplacental viviparous
**3**	*Galeorhinus galeus*	1	6.9	6.1	88.5	Aplacental viviparous
**4**	*Galeus melastomus*	10	8.2 ± 6.3	7.5 ± 5.9	88.7 ± 6	Oviparous
**5**	*Heptranchias perlo*	1	7.6	7.7	101.2	Aplacental viviparous
**6**	*Hexanchus griseus*	2	18.9 ± 2.6	16.4 ± 1.9	86.5 ± 1.7	Aplacental viviparous
**7**	*Carcharhinus macloti*	2	0.08 ± 0.04	0.07 ± 0.03	84.1 ± 2.3	Placental viviparous
**8**	*Carcharhinus melanopterus*	2	0.66 ± 0.5	0.57 ± 0.4	85 ± 2.2	Placental viviparous
**9**	*Carcharhinus sorrah*	7	0.25 ± 0.2	0.23 ± 0.2	94.8 ± 13.4	Placental viviparous
**10**	*Hemigaleus microstoma*	3	0.76 ± 0.3	0.76 ± 0.3	99.6 ± 13.4	Placental viviparous
**11**	*Mustelus mosis*	5	1.4 ± 1.8	1.3 ± 1.7	91.1 ± 5.4	Placental viviparous
**12**	*Rhizoprionodon acutus*	3	0.62 ± 0.3	0.56 ± 0.2	93.7 ± 10.7	Placental viviparous
**13**	*Sphyrna lewini*	7	4.3 ± 5.8	3.7 ± 4.4	94.2 ± 12.7	Placental viviparous
**14**	*Stegostoma fasciatum*	1	1.1	1.0	89.8	Oviparous
**15**	*Prionace glauca*	2	4.5 ± 0.9	4.0 ± 0.5	89.9 ± 5.3	Placental viviparous
**16**	*Isurus oxyrinchus*	2	3.0 ± 0.1	2.5 ± 0.1	83.0 ± 1.1	Aplacental viviparous (oophagous)
**17**	*Squalus acanthias*	2	0.75 ± 0.01	0.71 ± 0.01	95.0 ± 0.6	Aplacental viviparous
**18**	*Cetorhinus maximus*	4	0.09 ± 0.02	0.06 ± 0.01	64.8 ± 5.8	Aplacental viviparous (likely oophagous)

Table S4. The target hazard quotient (THQ) values and the estimated weekly intakes (EWIs) calculated for MeHg in samples from 18 shark species collected in the Mediterranean Sea, Atlantic and Indian Oceans.

Species	Mean MeHg as Hg	THQ		EWI
	mg kg^{-1} (ww)	Ef 365 days year^{-1}	Ef 48 days year^{-1}	mg kg^{-1} week^{-1}
Dalatias licha	1.90	21.6	2.8	0.0074
Etmopterus spinax	1.88	21.4	2.8	0.0073
Galeorhinus galeus	1.82	20.6	2.7	0.0071
Galeus melastomus	2.24	25.5	3.3	0.0087
Heptranchias perlo	2.30	26.1	3.4	0.0089
Hexanchus griseus	4.91	55.7	7.3	0.0190
C.macloti	0.02	0.23	0.03	0.0001
C. melanopterus	0.17	1.9	0.25	0.0007
C. sorrah	0.07	0.78	0.10	0.0003
Hemigaleus microstoma	0.23	2.6	0.34	0.0009
Mustelus mosis	0.38	4.3	0.56	0.0015
Rhizoprionodon acutus	0.17	1.9	0.25	0.0007
Sphyrna lewini	1.11	12.6	1.7	0.0043
Stegostoma fasciatum	0.30	3.4	0.45	0.0012
Prionace glauca	1.20	13.6	1.8	0.0047
Isurus oxyrinchus	0.76	8.6	1.1	0.0029
Squalus acanthias	0.21	2.4	0.32	0.0008
Cetorhinus maximus	0.02	0.19	0.03	0.0001

Table S5. Consumer risk categories based on allowable monthly fish consumption (CRmm), according to US EPA guideline (16 meals month^{-1} threshold).

Advisory class	Species	CRmm (meals/month)
**< 2 meals/month (very restricted consumption)**	*Hexanchus griseus*	0.19
	Heptranchias perlo	0.40
	Dalatias licha	0.50
	Galeorhinus galeus	0.50
	Prionace glauca	0.79
	Galeus melastomus	0.83
	Etmopterus spinax	1.02
	Isurus oxyrinchus	1.24
	Sphyrna lewini	1.41
**2–10 meals/month (controlled consumption)**	*Stegostoma fasciatum*	3.10
	Squalus acanthias	4.41
	Hemigaleus microstoma	4.88
	Rhizoprionodon acutus	6.21
	Mustelus mosis	6.47
	Carcharhinus melanopterus	7.92
**≥ 16 meals/month (generally safe consumption — EPA threshold)**	*Carcharhinus sorrah*	18.99
	Carcharhinus macloti	52.1
	Cetorhinus maximus	57.3

References

Adams, D.H., McMichael Jr, R.H., 1999. Mercury levels in four species of sharks from the Atlantic coast of Florida. Fish. Bull. 97, 372–379.

Adel, M., Copat, C., Reza, M., Asl, S., Oliveri, G., Babazadeh, M., Ferrante, M., 2018. Bioaccumulation of trace metals in banded Persian bamboo shark (*Chiloscyllium arabicum*) from the Persian Gulf: A food safety issue. Food Chem. Toxicol. 113, 198–203. https://doi.org/10.1016/j.fct.2018.01.027

Adel, M., Mohammadmoradi, K., Ley-Quiñonez, C.P., 2017. Trace element concentrations in muscle tissue of milk shark, (*Rhizoprionodon acutus*) from the Persian Gulf. Environ. Sci. Pollut. Res. 5933–5937. https://doi.org/10.1007/s11356-016-8358-6

Adel, M., Oliveri, G., Dadar, M., Mahjoub, M., Copat, C., 2016. Heavy metal concentrations in edible muscle of whitecheek shark, *Carcharhinus dussumieri* (elasmobranchii, chondrichthyes) from the Persian Gulf: A food safety issue. Food Chem. Toxicol. 97, 135–140. https://doi.org/10.1016/j.fct.2016.09.002

Al-Reasi, H.A., Ababneh, F.A., Lean, D.R., 2007. Evaluating mercury biomagnification in fish from a tropical marine environment using stable isotopes (δ13C and δ15N). Environ. Toxicol. Chem. 26, 1572–1581. https://doi.org/10.1897/06-359R.1

Al Ali, N.S., Malik, S., Muzaffar, S. Bin, 2025. Bioaccumulation of toxic elements in two shark species from the United Arab Emirates waters of the Arabian Gulf. Mar. Pollut. Bull. 219, 118344. https://doi.org/10.1016/j.marpolbul.2025.118344

Alencar Goyanna, F.A. De, Fernandes-Moises, B., Batista da Silva, G., Rezende, C.E. De, Rodrigues Bastos, W., Drude de Lacerda, L., 2025. Ecological drivers of mercury accumulation in oceanic apex predators: A human consumption advisory. Sci. Total. Environ. 970. https://doi.org/10.1016/j.scitotenv.2025.178994

Alves, L.M.F., Nunes, M., Marchand, P., Le, B., Mendes, S., Correia, J.P.S., Lemos, M.F.L., Novais, S.C., 2016. Science of the Total Environment Blue sharks (*Prionace glauca*) as bioindicators of pollution and health in the Atlantic Ocean: Contamination levels and biochemical stress responses. Sci. Total Environ. 563–564, 282–292. https://doi.org/10.1016/j.scitotenv.2016.04.085

Amorim-Lopes, C., Willmer, I.Q., Araujo, N.L.F., Pereira, L.H.S.D.S., Monteiro, F., Rocha, R.C.C., Pierre, T.D. Saint, Santos, L.N., Siciliano, S., Vianna, M., Hauser-davis, R.A., 2020. Mercury screening in highly consumed sharpnose sharks (*Rhizoprionodon lalandii and R. porosus*) caught artisanally in southeastern Brazil. Elem. Sci. Anthr. 1–13. https://doi.org/10.1525/elementa.022

Baeyens, W., Leermakers, M., Papina, T., Saprykin, A., Brion, N., Noyen, J., Gieter, M. De, Elskens, M., Goeyens, L., 2003. Environmental contamination and bioconcentration and biomagnification of mercury and methylmercury in North Sea and Scheldt Estuary Fish. Arch. Environ. Contam. Toxicol. 45, 498–508. https://doi.org/10.1007/s00244-003-2136-4

Baro-Camarasa, I., Marmolejo-Rodriguez, A.J., O'Hara, T.M., Castellini, J.M., Murillo-Cisneros, D.A., Martinez-Rincon, R.O., Elorriaga-Verplancken, F.R., Galvan-Magana, F., 2022. Mercury maternal transfer in two placental sharks and a yolk-sac ray from Baja California Sur, Mexico. Mar. Pollut. Bull. 179. https://doi.org/10.1016/j.marpolbul.2022.113672

Barros Paiva, R., Neves, A., Sequeira, V., Nunes, M.L., Serrano Gordo, L., Bandarra, N., 2012. Reproductive strategy of the female deep-water shark birdbeak dogfish, *Deania calcea*: lecithotrophy or matrotrophy? J. Mar. Biol. Assoc. United Kingdom 92, 387–394. https://doi.org/10.1017/S0025315411001743

Bendall, V.A., Barber, J.L., Papachlimitzou, A., Bolam, T., Warford, L., Hetherington, S.J., Silva, J.F., McCully, S.R., Losada, S., Maes, T., Ellis, J.R., Law, R.J., 2014. Organohalogen contaminants and trace metals in North-East Atlantic porbeagle shark (*Lamna nasus*). Mar. Pollut. Bull. 85, 280–286. https://doi.org/10.1016/j.marpolbul.2014.05.054

Bergées-Tiznado, M.E., Márquez-Farias, F., Lara-Mendoza, R.E., Torres-Rojas, Y.E., Galván-Magaña, F., Bojórquez-Leyva, H., Páez-Osuna, F., 2015. Mercury and selenium in muscle and target organs of scalloped hammerhead sharks *Sphyrna lewini* of the SE Gulf of California: dietary intake, molar ratios, loads, and human health risks. Arch. Environ. Contam. Toxicol. 69, 440–452. https://doi.org/10.1007/s00244-015-0226-8

Besnard, L., Le Crozier, G., Galvan-Magaña, F., Point, D., Kraffe, E., Ketchum, J., Martinez-Rincon, R.O., Schaal, G., 2021. Foraging depth depicts resource partitioning and contamination level in a pelagic shark assemblage: Insights from mercury stable isotopes. Environ. Pollut. 283. https://doi.org/10.1016/j.envpol.2021.117066

Besnard, L., Lucca, B.M., Shipley, O.N., Le Croizier, G., Martínez-Rincon, R.O., Sonke, J.E., Point, D., Magaña-Galván, F., Kraffe, E., Kwon, S.Y., Schaal, G., 2023. Mercury isotope clocks predict coastal residency and migration timing of hammerhead sharks. J. Appl. Ecol. 60, 803–813. https://doi.org/10.1111/1365-2664.14384

Biton-Porsmoguer, Sebastián, Banaru, D., Boudouresque, C.F., Dekeyser, I., Bouchoucha, M., Marco-Miralles, F., Lebreton, B., Guillou, G., Harmelin-vivien, M., 2018. Mercury in blue shark (*Prionace glauca*) and short fin mako (*Isurus oxyrinchus*) from north-eastern Atlantic: Implication for fishery management. Mar. Pollut. Bull. 127, 131–138. https://doi.org/10.1016/j.marpolbul.2017.12.006

Biton-Porsmoguer, S., Marco-Miralles, F., Bouchoucha, M., 2024. Mercury levels in tissues (cartilage, skin, and muscle) of the Greenland shark (*Somniosus microcephalus*): Potential contamination sources and implications for health and conservation. Polar Sci. 42, 101079. https://doi.org/10.1016/j.polar.2024.101079

Blanco, S.L., González, J.C., Vieites, J.M., 2009. Food Additives & Contaminants: Part B: Surveillance Mercury, cadmium and lead levels in samples of the main traded fish and shellfish species in Galicia, Spain View Dataset. Food Addit. Contam. 1, 37–41. https://doi.org/10.1080/19393210802236893

Boldrocchi, G., Monticelli, D., Omar, Y.M., Bettinetti, R., 2019. Trace elements and POPs in two commercial shark species from Djibouti: Implications for human exposure. Sci. Total Environ. 669, 637–648. https://doi.org/10.1016/j.scitotenv.2019.03.122

Boldrocchi, G., Spanu, D., Mazzoni, M., Omar, M., Baneschi, I., Boschi, C., Zinzula, L., Bettinetti, R., Monticelli, D., 2021. Bioaccumulation and biomagnification in elasmobranchs: A concurrent assessment of trophic transfer of trace elements in 12 species from the Indian Ocean. Mar. Pollut. Bull. 172, 112853. https://doi.org/10.1016/j.marpolbul.2021.112853

Boldrocchi, G., Spanu, D., Polesello, S., Valsecchi, S., Garibaldi, F., Lanteri, L., Ferrario, C., Monticelli, D., Bettinetti, R., 2022. Legacy and emerging contaminants in the endangered filter feeder basking shark *Cetorhinus maximus*. Mar. Pollut. Bull. 176, 113466. https://doi.org/10.1016/j.marpolbul.2022.113466

Bosch, A.C., O'Neill, B., Sigge, G.O., Kerwath, S.E., Hoffman, L.C., 2016. Heavy metal accumulation and toxicity in smoothhound (*Mustelus mustelus*) shark from Langebaan Lagoon, South Africa. FOOD Chem. 190, 871–878. https://doi.org/10.1016/j.foodchem.2015.06.034

Branco, V., Vale, C., Canario, J., Neves dos Santos, M., 2007. Mercury and selenium in blue shark (*Prionace glauca*, *L.* 1758) and swordfish (*Xiphias gladius*, *L.* 1758) from two areas of the Atlantic Ocean. Environ. Pollut. 150, 373–380. https://doi.org/10.1016/j.envpol.2007.01.040

Branco, V., Vale, C., Canario, J., Raimundo, J., 2004. Total and organic mercury concentrations in muscle tissue of the blue shark (*Prionace glauca*) from the north-east Atlantic. Mar. Pollut. Bull. 49, 854–874. doi: 10.1016/j.marpolbul.2004.09.002.

Bryhim, J.R.O., Adams, D.H., Spaet, J.L.Y., Mills, G., Lance, S.L., 2017. Relationships of mercury concentrations across tissue types, muscle regions and fi ns for two shark species. Environ. Pollut. 223, 323–333. https://doi.org/10.1016/j.envpol.2017.01.029

Burger, J., Gochfeld, M., 2011. Mercury and selenium levels in 19 species of saltwater fish from New Jersey as a function of species, size, and season. Sci. Total Environ. 409, 1418–1429. https://doi.org/10.1016/j.scitotenv.2010.12.034

Cagnazzi, D., Broadhurst, M.K., Reichelt-Brushett, A., 2019. Metal contamination among endangered, threatened and protected marine vertebrates o ff south-eastern Australia. Ecol. Indic. 107, 105658. https://doi.org/10.1016/j.ecolind.2019.105658

Cai, Y., Rooker, J.R., Gill, G.A., Turner, J.P., 2007. Bioaccumulation of mercury in pelagic fishes from the northern Gulf of Mexico. Can. J. Fish. Aquat. Sci. 469, 458–469. https://doi.org/10.1139/F07-017

Campos, A.S., Bezerra, M.F., Moura, V.L., Faria, V. V, Rezende, C.E., Bastos, W.R., Oliveira, I.A.S., Lacerda, L.D., 2024. Assessment of mercury bioaccumulation in a tropical elasmobranch assemblage. Environ. Chem. 21, EN24065. https://doi.org/10.1071/EN24065

Chouvelon, T., Cresson, P., Bouchoucha, M., Brach-papa, C., Bustamante, P., Crochet, S., Marco-Miralles, F., Thomas, B., Knoery, J., 2018. Oligotrophy as a major driver of mercury bioaccumulation in medium-to high-trophic level consumers: A marine ecosystem-comparative study. Environ. Pollut. 233, 844–854. https://doi.org/10.1016/j.envpol.2017.11.015

Chouvelon, T., Spitz, J., Caurant, F., Mendez-Fernandez, P., Autier, J., Chappuis, A., Bustamante, P., 2012. Enhanced bioaccumulation of mercury in deep-sea fauna from the Bay of Biscay (north-east Atlantic) in relation to trophic positions identified by analysis of carbon and nitrogen stable isotopes. Deep. Res. Part I 65, 113–124. https://doi.org/10.1016/j.dsr.2012.02.010

Coelho, J.P., Santos, H., Reis, A.T., Falcão, J., Rodrigues, E.T., Pereira, M.E., Duarte, A.C., Pardal, M.A., 2010. Mercury bioaccumulation in the spotted dogfish (*Scyliorhinus canicula*) from the Atlantic Ocean. Mar. Pollut. Bull. 60, 1372–1375. https://doi.org/10.1016/j.marpolbul.2010.05.008

Corsolini, S., Ancora, S., Bianchi, N., Mariotti, G., Leonzio, C., Christiansen, J.S., 2014. Organotropism of persistent organic pollutants and heavy metals in the Greenland shark *Somniosus microcephalus* in NE Greenland. Mar. Pollut. Bull. 87, 381–387. https://doi.org/10.1016/j.marpolbul.2014.07.021

Crawford, L.M., Gelsleichter, J., Newton, A.L., Hoopes, L., Lee, C., Fisher, N.S., Adams, D.H., Giraudo, M., Mcelroy, A.E., 2023. Associations between total mercury, trace minerals, and blood health markers in Northwest Atlantic white sharks (*Carcharodon carcharias*). Mar. Pollut. Bull. 195, 115533. https://doi.org/10.1016/j.marpolbul.2023.115533

Cresson, P., Fabri, M.C., Bouchoucha, M., Brach Papa, C., Chavanon, F., Jadaud, A., Knoery, J., Miralles, F., Cossa, D., 2014. Mercury in organisms from the Northwestern Mediterranean slope: Importance of food sources. Sci. Total Environ. 497–498, 229–238. https://doi.org/10.1016/j.scitotenv.2014.07.069

Croizier, L., Lorrain, A., Sonke, J.E., Hoyos-Padilla, E.M., Galva, F., Santana-Morales, O., Aquino-Baleyto, M., Becerril-Garcia, E.E., Ketchum, J., Block, B., Carlisle, A., Jorgensen, S.J., Besnard, L., Jung, A., Schaal, G., Point, D., 2020. The twilight zone as a major foraging habitat and mercury source for the great white shark. Environ. Sci. Technol. 54, 15872–15882. https://doi.org/10.1021/acs.est.0c05621

Davis, J.A., Ross, J.R.M., Bezalel, S., Sim, L., Bonnema, A., Ichikawa, G., Heim, W.A., Schiff, K., Eagles-smith, C.A., Ackerman, J.T., 2016. Hg concentrations in fish from coastal waters of California and Western North America. Sci. Total Environ. 568, 1146–1156. https://doi.org/10.1016/j.scitotenv.2016.03.093

de Moura, J.F., Merico, A., Montone, R.C., Silva, J., Seixas, T.G., Marcus, J., De Oliveira Godoy, J.M., Dillenburg, T., Hauser-Davis, R.A., Madeira di Beneditto, A.P., Cardinot, E., Castro, D., Soledade, L., Siciliano, S., 2015. Assessment of trace elements, POPs, 210 Po

and stable isotopes (15N and 13C) in a rare filter-feeding shark: The megamouth. Mar. Pollut. Bull. 95, 402–406. https://doi.org/10.1016/j.marpolbul.2015.03.038

Dutton, J., Hobbs, J.C., Joung, S., Schmidt, J. V, 2023. Mercury Concentrations in Whale Shark (*Rhincodon typus*) Embryo Muscle Tissue. Bull. Environ. Contam. Toxicol. 1–6.

Dutton, J., Venuti, V.M., 2019. Comparison of Maternal and Embryonic Trace Element Concentrations in Common Thresher Shark (*Alopias vulpinus*) Muscle Tissue. Bull. Environ. Contam. Toxicol. 103, 380–384. https://doi.org/10.1007/s00128-019-02667-1

Ehnert-Russo, S.., Gelsleichter, J., 2020. Mercury Accumulation and Effects in the Brain of the Atlantic Sharpnose Shark (*Rhizoprionodon terraenovae*). Arch. Environ. Contam. Toxicol. 78, 267–283. https://doi.org/10.1007/s00244-019-00691-0

Elsayed, H., Yigiterhan, O., Al-ansari, E.M.A.S., Al-ashwel, A.A., 2020. Methylmercury bioaccumulation among different food chain levels in the EEZ of Qatar (Arabian Gulf). Reg. Stud. Mar. Sci. 37, 101334. https://doi.org/10.1016/j.rsma.2020.101334

Endo, T., Hisamichi, Y., Haraguchi, K., Kato, Y., Ohta, C., Koga, N., 2008. Hg, Zn and Cu levels in the muscle and liver of tiger sharks (*Galeocerdo cuvier*) from the coast of Ishigaki Island, Japan: Relationship between metal concentrations and body length. Mar. Pollut. Bull. 56, 1774–1780. https://doi.org/10.1016/j.marpolbul.2008.06.003

Endo, T., Hisamichi, Y., Kimura, O., Kotaki, Y., Kato, Y., Ohta, C., Koga, N., Haraguchi, K., 2009. Contamination levels of mercury in the muscle of female and male spiny dogfishes (*Squalus acanthias*) caught off the coast of Japan. Chemosphere 77, 1333–1337. https://doi.org/10.1016/j.chemosphere.2009.09.041

Endo, T., Hisamichi, Y., Kimura, O., Ogasawara, H., 2013. Levels of Mercury in Muscle and Liver of Star-Spotted Dogfish (*Mustelus manazo*) from the Northern Region of Japan: A Comparison with Spiny Dogfish (*Squalus acanthias*). Arch. Environ. Contam. Toxicol. 64, 467–474. https://doi.org/10.1007/s00244-012-9858-0

Endo, T., Kimura, O., Ohta, C., Koga, N., Kato, Y., 2016. Metal concentrations in the liver and stable isotope ratios of carbon and nitrogen in the muscle of silvertip shark (*Carcharhinus albimarginatus*) Culled off Ishigaki Island, Japan: Changes with Growth. PLoS One 11, 1–18. https://doi.org/10.1371/journal.pone.0147797

Escobar-Sánchez, O., Galván-Magaña, F., Rosiles-Martínez, R., 2011. Biomagnification of mercury and selenium in blue shark *Prionace glauca* from the Pacific Ocean off Mexico. Biol. Trace Elem. Res. 144, 550–559. https://doi.org/10.1007/s12011-011-9040-y

Escobar-Sánchez, O., Galván-Magaña, F., Rosiles-Martínez, R., 2010. Mercury and selenium bioaccumulation in the smooth hammerhead shark, *Sphyrna zygaena Linnaeus*, from the Mexican Pacific Ocean. Bull. Environ. Contam. Toxicol. 84, 488–491. https://doi.org/10.1007/s00128-010-9966-3

Ferreira, A.G., Faria, V.V., Veiga de Carvalho, C.E., Teixeira Lessa, R.P., Marcante Santana da Silva, F., 2004. Total mercury in the night shark, *Carcharhinus signatus* in the

Western Equatorial Atlantic Ocean. Brazilian Arch. Biol. Technol. 47, 629–634. https://doi.org/10.1590/S1516-89132004000400016

Forsyth, D.S., Casey, V., Dabeka, R.W., Mckenzie, A., 2007. Methylmercury levels in predatory fish species marketed in Canada. Food Addit. Contam. 21, 37–41. https://doi.org/10.1080/02652030400004259

Frias-Espericueta, M.G., Ruelas-inzunza, J., Benitez-Lizarraga, R., Escobar-Sánchez, O., Osuna-Martinez, C., Delgado-Alvarez, C.G., Aguilar-Juarez, M., Osuna-Lopez, I., Voltolina, D., 2019. Risk assessment of mercury in sharks (*Rhizoprionodon longurio*) caught in the coastal zone of Northwest Mexico. J. Consum. Prot. Food Saf. 6, 349–354. https://doi.org/10.1007/s00003-019-01232-6

Frías-espericueta, M.G., Zamora-sarabia, F.K.G., Márquez-Farías, J.F., Ruelas-inzunza, J., Voltolina, D., 2015. Total mercury in female Pacific sharpnose sharks *Rhizoprionodon longurio* and their embryos. Lat. Am. J. Aquat. Res. 43, 534–538. https://doi.org/10.3856/vol43-issue3-fulltext-14

Gaion, A., Scuderi, A., Sartori, D., Pellegrini, D., Ligas, A., 2016. Trace metals in tissues of *Galeus melastomus Rafinesque*, 1810 from the northern Tyrrhenian Sea (NW Mediterranean). Acta Adriat. 57, 165–172.

Gallo, S., Leonetti, F.L., Reinero, F.R., Micarelli, P., Passarelli, L., Giglio, G., Milazzo, C., Imbrogno, S., Barca, D., Bottaro, M., Sperone, E., 2025. Bioaccumulation patterns in different tissues of twelve species of elasmobranchs from the Tyrrhenian and Ionian Sea (Calabria). Environments 12, 1–16. https://doi.org/10.3390/ environments12010012

García-Hernández, J., Cadena-cárdenas, L., García-de-la-parra, L.M., Márquez-Farías, F., 2007. Total mercury content found in edible tissues of top predator fish from the Gulf of California, Mexico. Toxicol. Environ. Chem. 37–41. https://doi.org/10.1080/02772240601165594

Gelsleichter, J., Sparkman, G., Howey, L.A., Brooks, E.J., Shipley, O.N., 2020. Elevated accumulation of the toxic metal mercury in the Critically Endangered oceanic whitetip shark *Carcharhinus longimanus* from the northwestern Atlantic Ocean. Endanger. Species Res. 43, 267–279. https://doi.org/10.3354/esr01068

Gilbert, J.M., Reichelt-Brushett, A.J., Butcher, P.A., Mcgrath, S.P., Peddemors, V.M., Bowling, A.C., Christidis, L., 2015. Metal and metalloid concentrations in the tissues of dusky *Carcharhinus obscurus,* sandbar *C. plumbeus* and white *Carcharodon carcharias* sharks from south-eastern Australian waters, and the implications for human consumption. Mar. Pollut. Bull. 92, 186–194. https://doi.org/10.1016/j.marpolbul.2014.12.037

Giovos, I., Ciprian, M., Copat, C., Felici, A., Katselis, G., Kazlari, Z., Loukovitis, D., Mazzoldi, C., Naasan, R., Sanchez-Rea, A., Tiralongo, F., Moutopoulos, D., 2025. Trace element levels in the sharks and rays of Amvrakikos Wetlands. Sci. Total Environ. 1002, 1–11. https://doi.org/10.1016/j.scitotenv.2025.180577

Greenfield, B.K., Davis, J.A., Fairey, R., Roberts, C., Crane, D., Ichikawa, G., 2005. Seasonal, interannual, and long-term variation in sport fish contamination, San Francisco Bay. Sci. Total Environ. 336, 25–43. https://doi.org/10.1016/j.scitotenv.2004.05.023

Greig, R., Wenztoff, D., Shelpuk, C., 1975. Mercury concentrations in fish, North Atlantic Offshore Waters-1971. Pestic. Monit. J. 9.

Gustinelli Arantes de Carvalho, G., Manoel Degaspari, I.A., Branco, V., Canario, J., Ferreira de Amorim, A., Kennedy, V.H., Ferreira, J.R., 2014. Assessment of total and organic mercury levels in blue sharks (*Prionace glauca*) from the South and Southeastern Brazilian Coast. Biol. Trace Elem. Res. 159, 128–134. https://doi.org/10.1007/s12011-014-9995-6

Hammerschlag, N., Davis, D.A., Mondo, K., Seely, M.S., Murch, S.J., Glover, W.B., Divoll, T., Evers, D.C., Mash, D.C., 2016. Cyanobacterial neurotoxin bmaa and mercury in sharks. Toxins (Basel). 8, 1–14. https://doi.org/10.3390/toxins8080238

Heidarieh, H., Gholamhosseini, A., Soltanian, S., Heidarieh, M., 2025. Assessment of heavy metals concentration and health risk in four species of Persian Gulf sharks in Hormozgan Province. J. Trace Elem. Med. Biol. 87, 127581. https://doi.org/10.1016/j.jtemb.2024.127581

Higueruelo, A., Besada, V., Sanchez-Marin, P., Muns-Pujadas, L., Constenla, M., Dallares, S., Carreras-Colom, E., Rodriguez-Romeu, O., Soler-Membrives, A., 2025. A “toxic trio” (mercury, lead and cadmium) metal assessment in marine commercial species from Northwestern Mediterranean Sea: risk and recommendations. Environ. Res. 282. https://doi.org/10.1016/j.envres.2025.122022

Hornung, H., Krom, M.D., Cohen, Y., Bernhard, M., 1993. Trace metal content in deep-water sharks from the eastern Mediterranean Sea. Mar. Biol. 115, 331–338. https://doi.org/10.1007/BF00346351

Hueter, R.E., Fong, W.G., Henderson, G., French, M.F., Manire, C.A., 1995. Methylmercury concentration in shark muscle by species size and distribution of sharks in Florida coastal waters. Water, Air Soil Pollut. 80, 893–899. https://doi.org/10.1007/BF01189741

Hurtado-banda, R., Gómez-Álvarez, A., Márquez-Farias, J.F., Córdoba-Figueroa, M., Navarro-García, G., Medina-Juárez, L.A., 2012. Total mercury in liver and muscle tissue of two coastal sharks from the northwest of Mexico. Bull. Environ. Contam. Toxicol. 88, 971–975. https://doi.org/10.1007/s00128-012-0623-x

Julio, T.G., Moura, V.L., Lacerda, L.D., Lessa, R.P.T., 2022. Mercury concentrations in coastal Elasmobranchs (*Hypanus guttatus* and *Rhizoprionodon porosus*) and human exposure in Pernambuco, Northeastern Brazil. An. Acad. Bras. Cienc. 94, 1–14. https://doi.org/10.1590/0001-3765202220220045

Kaneko, J.J., Ralston, N.V.C., 2007. Selenium and Mercury in Pelagic Fish in the Central North Pacific Near Hawaii. Biol. Trace Elem. Res. 119, 242–254. https://doi.org/10.1007/s12011-007-8004-8

Karimi, R., Frisk, M., Fisher, N.S., 2013. Contrasting food web factor and body size relationships with Hg and Se concentrations in marine biota. PLoS One 8. https://doi.org/10.1371/journal.pone.0074695

Kim, S.W., Han, S.J., Kim, Yonggab, Jun, J.W., Giri, S.S., Chi, C., Yun, S., Kim, H.J., Kim, S.G., Kang, J.W., Kwon, J., Oh, T., Cha, J., Han, S., Lee, B.C., Park, T., Kim, Yeop, Chang, S., Id, P., 2019. Heavy metal accumulation in and food safety of shark meat from Jeju Island, Republic of Korea. PLoS One 1–18. https://doi.org/10.1371/journal.pone.0212410

Kiszka, J.J., Aubail, A., Hussey, N.E., Heithaus, M.R., Caurant, F., Bustamante, P., 2015. Plasticity of trophic interactions among sharks from the oceanic south-western Indian Ocean revealed by stable isotope and mercury analyses. Deep. Res. Part I 96, 49–58. https://doi.org/10.1016/j.dsr.2014.11.006

Kousteni, V., Megalofonou, P., Dassenakis, M., Stathopoulou, E., 2006. Total mercury concentrations in edible tissues of two elasmobranch species from Crete (eastern Mediterranean Sea). Cybium 30, 119–123. https://doi.org/10.26028/cybium/2006-304supp-016

Lacerda, L.., Paraquetti, H.H.M., Marins, R.., Rezende, C.., Zalmon, I.., Gomes, M.P., Farias, V., 2000. Mercury content in shark species from the south-eastern Brazilian coast. Rev. Bras. Biol. 60, 571–576. https://doi.org/10.1590/S0034-71082000000400005

Li, Z., Pethybridge, H.R., Gong, Y., Wu, F., Dai, X., Li, Y., 2022. Effect of body size, feeding ecology and maternal transfer on mercury accumulation of vulnerable silky shark *Carcharhinus falciformis* in the eastern tropical pacific. Environ. Pollut. 309, 119751. https://doi.org/10.1016/j.envpol.2022.119751

Li, Z., Pethybridge, H.R., Wu, F., Li, Y., 2023. Mercury bioaccumulation in thresher sharks from the eastern tropical Pacific: Influences of body size, maturation stage, and feeding habitat. Sci. Total Environ. 872, 162248. https://doi.org/10.1016/j.scitotenv.2023.162248

Liu, B., Chen, I., Chen, P., Chen, T., Hwang, D., 2023. Risk assessment of methylmercury and species identification in shark meats ingested by Taiwan children. Food Control 145, 109461. https://doi.org/10.1016/j.foodcont.2022.109461

Lloret-lloret, E., Fernandez, A., Navarro, J., Bravo, A.G., Sanpera, C., Esteban, A., Bellido, J.M., Coll, M., Gimenez, J., 2025. Understanding the role of biological, environmental and human-impact factors on mercury concentrations in a demersal mesopredator shark. Mar. Pollut. Bull. 220. https://doi.org/10.1016/j.marpolbul.2025.118358

Loose, E. De, Gayford, J.H., Karalic, E., Annibaldi, A., Girolametti, F., Truzzi, C., Illuminati, S., Besirovic, H., Gaji, A.A., 2025. Trace element concentration and toxicity in blackspotted smooth-hound sharks (*Mustelus punctulatus*) from the southern Adriatic Sea: Implications

for consumer safety. Mar. Pollut. Bull. 213. https://doi.org/10.1016/j.marpolbul.2025.117630

Lopez, S.A., Abarca, N.L., Melendez, R., 2013. Heavy metal concentrations of two highly migratory sharks (*Prionace glauca* and *Isurus oxyrinchus*) in the southeastern Pacific waters: comments on public health and conservation. Trop. Conserv. Sci. 6, 126–137. https://doi.org/10.1177/194008291300600103

Lyons, K., Carlisle, A., Preti, A., Mull, C., Blasius, M., Sullivan, J.O., Winkler, C., Lowe, C.G., 2013. Effects of trophic ecology and habitat use on maternal transfer of contaminants in four species of young of the year lamniform sharks. Mar. Environ. Res. 90, 27–38. https://doi.org/10.1016/j.marenvres.2013.05.009

Lyons, K., Lowe, C.G., 2013. Mechanisms of maternal transfer of organochlorine contaminants and mercury in the common thresher shark (*Alopias vulpinus*). Can. J. Fish. Aquat. Sci. 70, 1667–1672. https://doi.org/10.1139/cjfas-2013-0222

Marsico, E.., Machado, M.E.., Knoff, M., Sao Clemente, S.., 2007. Total mercury in sharks along the southern Brazilian Coast. Arq. Bras. Med. Vet. e Zootec. 59, 1593–1596.

Matos, J., Lourenço, H.M., Brito, P., Maulvault, A.L., Martins, L.L., Afonso, C., 2015. In fluence of bioaccessibility of total mercury, methylmercury and selenium on the risk / benefit associated to the consumption of raw and cooked blue shark (*Prionace glauca*). Environ. Res. 143, 123–129. https://doi.org/10.1016/j.envres.2015.09.015

Matulik, A.G., Kerstetter, D.W., Hammerschlag, N., Divoll, T., Hammerschmidt, C.R., Evers, D.C., 2017. Bioaccumulation and biomagnification of mercury and methylmercury in four sympatric coastal sharks in a protected subtropical lagoon. Mar. Pollut. Bull. 116, 357–364. https://doi.org/10.1016/j.marpolbul.2017.01.033

Maurice, L., Le Croizier, G., Morales, G., Carpintero, N., Guayasamin, J.M., Sonke, J., Paez-Rosas, D., Point, D., Bustos, W., Ochoa-herrera, V., 2021. Ecotoxicology and Environmental Safety Concentrations and stable isotopes of mercury in sharks of the Galapagos Marine Reserve: Human health concerns and feeding patterns. Ecotoxicol. Environ. Saf. 215. https://doi.org/10.1016/j.ecoenv.2021.112122

Maynard, G.A., Baumann, Z., 2020. Methylmercury levels in commercially harvested spiny dogfish captured off the coast of Massachusetts. Trans. Am. Fish. Soc. 149, 486–497. https://doi.org/10.1002/tafs.10243

Maz-Courrau, A., López-Vera, C., Galván-Magaña, F., Escobar-Sánchez, O., Rosiles-Martínez, R., Sanjuan-Muñoz, A., 2012. Bioaccumulation and biomagnification of total mercury in four exploited shark species in the Baja California Peninsula, Mexico. Bull. Environ. Contam. Toxicol. 88, 129–134. https://doi.org/10.1007/s00128-011-0499-1

McKinney, M.A., Dean, K., Hussey, N.E., Cliff, G., Wintner, S.P., Dudley, S.F.J., Zungu, M.P., Fisk, A.T., 2016. Global versus local causes and health implications of high mercury concentrations in sharks from the east coast of South Africa. Sci. Total Environ. 541, 176–183. https://doi.org/10.1016/j.scitotenv.2015.09.074

McMeans, B.C., Arts, M.T., Fisk, A.T., 2015. Impacts of food web structure and feeding behaviour on mercury exposure in Greenland Sharks (*Somniosus microcephalus*). Sci. Total Environ. 509–510, 216–225. https://doi.org/10.1016/j.scitotenv.2014.01.128

Medina-Morales, S.A., Corro-Espinosa, D., Escobar-Sánchez, O., Delgado-Álvarez, C.G., Ruelas-Inzunza, J., Frías-Espericueta, M.G., Jara-Marini, M.E., Páez-osuna, F., 2020. Mercury (Hg) and selenium (Se) content in the shark *Mustelus henlei* (*Triakidae*) in the northern Mexican Pacific. Environ. Sci. Pollut. Res. 27, 16774–16783. https://doi.org/10.1007/s11356-020-08198-1

Mohammed, A., Mohammed, T., 2017. Mercury, arsenic, cadmium and lead in two commercial shark species (*Sphyrna lewini* and *Caraharinus porosus*) in Trinidad and Tobago. Mar. Pollut. Bull. 119, 214–218. https://doi.org/10.1016/j.marpolbul.2017.04.025

Mol, J.H., Ramlal, J.S., Lietar, C., Verloo, M., 2001. Mercury contamination in freshwater, estuarine, and marine fishes in relation to small-scale gold mining in Suriname, South America. Environ. Res. Sect. A 86, 183–197. https://doi.org/10.1006/enrs.2001.4256

Moore, A.B.M., Bolam, T., Lyons, B.P., Ellis, J.R., 2015. Concentrations of trace elements in a rare and threatened coastal shark from the Arabian Gulf (smoothtooth blacktip *Carcharhinus leiodon*). Mar. Pollut. Bull. 100, 646–650. https://doi.org/10.1016/j.marpolbul.2015.06.005

Monticelli, D., Castelletti, A., Civati, D., Recchia, S. & Dossi, C. How to Efficiently Produce Ultrapure Acids. International Journal of Analytical Chemistry 2019, 8–11 (2019).

Mull, C., O'Sullivan, J., Lowe, C.G., 2012. Heavy metals, trace elements, and organochlorine contaminants in muscle and liver tissue of juvenile white sharks, *Carcharodon carcharias*, from the Southern California Bight. Glob. Perspect. Biol. life Hist. white shark. https://doi.org/10.1201/b11532-7

Nam, D.-H., Adams, D.H., Reyier, E.A., Basu, N., 2011. Mercury and selenium levels in lemon sharks (*Negaprion brevirostris*) in relation to a harmful red tide event. Environ. Monit. Assess. 176, 549–559. https://doi.org/10.1007/s10661-010-1603-4

Nicolaus, E.E.M., Bendall, V.A., Bolam, T.P.C., Maes, T., Ellis, J.R., 2016. Concentrations of mercury and other trace elements in porbeagle shark *Lamna nasus*. Mar. Pollut. Bull. 112, 406–410. https://doi.org/10.1016/j.marpolbul.2016.07.047

Núñez-Nogueira, G., 2005. Concentration of essential and non-essential metals in two shark species commonly caught in Mexican (Gulf of Mexico), in: Golfo de Mexico Contaminacion e Impacto Ambiental: Diagnostico y Tendencias.

Ouedraogo, O., Amyot, M., 2011. Effects of various cooking methods and food components on bioaccessibility of mercury from fish. Environ. Res. 111, 1064–1069. https://doi.org/10.1016/j.envres.2011.09.018

Pantoja-Echevarría, L.M., Marmolejo-Rodriguez, A.J., Galván-Magaña, F., Elorriaga-Verplancken, F.R., Tripp-Valdez, A., Tamburin, E., Lara, A., Jonathan, M.P., Sujitha, S.B., Arreola-Mendoza, L., 2021. Mercury and selenium concentrations in different tissues of

brown smooth-hound shark (*Mustelus henlei*) from the western coast of Baja California Sur, Mexico. Mar. Pollut. Bull. 170. https://doi.org/10.1016/j.marpolbul.2021.112609

Pantoja-Echevarría, L.M., Marmolejo-Rodriguez, A.J., Galván-Magaña, F., Elorriaga-Verplancken, F.R., Tripp-Valdez, A., Tamburin, E., Lara, A., Jonathan, M.P., Sujitha, S.B., Delgado-Huertas, A., Arreola-Mendoza, L., 2023. Trophic structure and biomagnification of cadmium, mercury and selenium in brown smooth hound shark (*Mustelus henlei*) within a trophic web. Food Webs 34. https://doi.org/10.1016/j.fooweb.2022.e00263

Pantoja-Echevarría, L.M., Marmolejo-Rodriguez, A.J., Galván-Magaña, F., Elorriaga-verplancken, F.R., Tripp-Valdez, A., Tamburin, E., Lara, A., Muthuswamy, J., Suresh, S., Pintueles-Tamayo, J., Valiente, S., Arreola-Mendoza, L., 2024. Risk assessment for shark consumers exposed to mercury, selenium and cadmium in Mexico. Mar. Pollut. Bull. 209, 4–9. https://doi.org/10.1016/j.marpolbul.2024.117205

Pethybridge, H., Butler, E.C. V, Cossa, D., Daley, R., 2012. Trophic structure and biomagnification of mercury in an assemblage of deepwater chondrichthyans from southeastern Australia. Mar. Ecol. Prog. Ser. 451, 163–174. https://doi.org/10.3354/meps09593

Pethybridge, H., Cossa, D., Butler, E.C. V, 2010. Mercury in 16 demersal sharks from southeast Australia: Biotic and abiotic sources of variation and consumer health implications. Mar. Environ. Res. 69, 18–26. https://doi.org/10.1016/j.marenvres.2009.07.006

Pinho, A.P. De, Dave, J.R., Martins, A.S., Costa, P.A.S., Olavo, G., Valentin, J., 2002. Total mercury in muscle tissue of five shark species from brazilian offshore waters: effects of feeding habit, sex, and length. Environ. Res. Sect. A 89, 250–258. https://doi.org/10.1006/enrs.2002.4365

Pinho, J.V. De, Willmer, I.Q., Lopes, A.P., Fonsêca, R., Dillenburg, T., Charvet, P., Gomes, A., Hauser-Davis, R.A., 2025. Metallic makos: metal and metalloid levels and human health risks arising from the consumption of shortfin makos (*Isurus oxyrinchus*) from Southeastern Brazil. Biol. Trace Elem. Res. 203, 5401–5415. https://doi.org/10.1007/s12011-025-04572-7

Powell, J.H., Powell, R.E., 2001. Trace elements in fish overlying subaqueous tailings in the tropical west pacific. Water, Air Soil Pollut. 125, 81–104. https://doi.org/10.1023/A:1005211832691

Rafael, T., Laura, A., Felipe, G., Sujitha, S.B., Jonathan, M.P., 2019. Understanding the antagonism of Hg and Se in two shark species from Baja California South, México. Sci. Total Environ. 650, 202–209. https://doi.org/10.1016/j.scitotenv.2018.08.261

Rechimont, M.E., Amezcua, F., Ruelas-Inzunza, J.R., Cruz-García, R., Vallarta-Zárate, J.R.F., Amezcua-Linares, F., 2025. Mercury and selenium trophic transfer in the Mexican California current ecosystem using a top predator as a model. Fishes 10, 1–13. https://doi.org/10.3390/ fishes10060275

Reinero, F.R., Sperone, E., Gallo, S., Barca, D., Leonetti, F.L., Giglio, G., Micarelli, P., 2025. First insights into bioaccumulation patterns in different tissues of the greenland shark *Somniosus microcephalus* from Kulusuk (Southeastern Greenland). Biology (Basel). 14, 1–15. https://doi.org/ 10.3390/biology14070857

Reistad, N.A., Norris, S.B., Rumbold, D.G., 2021. Mercury in neonatal and juvenile blacktip sharks (*Carcharhinus limbatus*). Part I: exposure assessment. Ecotoxicology 187–197. https://doi.org/10.1007/s10646-020-02322-0

Riesgo, L., Sanpera, C., Garcia-Barcelona, S., Sanchez-Fortun, M., Coll, M., Navarro, J., 2023. Understanding the role of ecological factors affecting mercury concentrations in the blue shark (*Prionace glauca*). Chemosphere 313. https://doi.org/10.1016/j.chemosphere.2022.137642

Rodrigues, A.C.M., Gravato, C., Galvao, D., Silva, V.S., M.V, A., Soares, M., Gonçalves, J.M.S., Ellis, J.R., Vieira, R.P., 2022. Ecophysiological effects of mercury bioaccumulation and biochemical stress in the deep-water mesopredator *Etmopterus spinax* (*Elasmobranchii*; *Etmopteridae*). J. Hazard. Mater. 423. https://doi.org/10.1016/j.jhazmat.2021.127245

Rodríguez-Gutiérrez, J., Galván-magaña, F., Jacobo-estrada, T., 2020. Mercury – selenium concentrations in silky sharks (*Carcharhinus falciformis*) and their toxicological concerns in the southern Mexican Pacific. Mar. Pollut. Bull. 153, 111011. https://doi.org/10.1016/j.marpolbul.2020.111011

Roubie, E., Karavoltsos, S., Sakellari, A., Katsikatsos, N., Dassenakis, M., Megalofonou, P., 2024. Trace metals distribution in tissues of 10 different shark species from the eastern Mediterranean Sea. Fishes 9. https://doi.org/10.3390/ fishes9020077

Ruelas, M.E.R.J., Amezcua, I.F., Osuna, F.P., Géniz, J.L.C., 2024. Hg and Se in muscle and liver of blue shark (*Prionace glauca*) from the entrance of the Gulf of California: An insight to the potential risk to human health. Arch. Environ. Contam. Toxicol. 86, 165–177. https://doi.org/10.1007/s00244-024-01054-0

Rumbold, D., Wasno, R., Hammerschlag, N., Volety, A., 2014. Mercury accumulation in sharks from the coastal waters of southwest Florida. Arch. Environ. Contam. Toxicol. 67, 402–412. https://doi.org/10.1007/s00244-014-0050-6

Sadhu, A.K., Kim, J.P., Furrell, H., Bostock, B., 2015. Methylmercury concentrations in edible fish and shellfish from Dunedin, and other regions around the South Island, New Zealand. Mar. Pollut. Bull. 101, 386–390. https://doi.org/10.1016/j.marpolbul.2015.10.013

Sandoval-Herrera, N.I., Vargas-Soto, J.S., Espinoza, M., Clarke, T.M., Fisk, A.T., Wehrtmann, I.S., 2016. Mercury levels in muscle tissue of four common elasmobranch species from the Pacific coast of Costa Rica, Central America. Reg. Stud. Mar. Sci. 3, 254–261. https://doi.org/10.1016/j.rsma.2015.11.011

Sisma-Ventura, G., Silverman, J., Segal, Y., Hauzer, H., Khadra, M.A., Stern, N., Guy-Haim, T., Herut, B., 2024. Exceptionally high levels of total mercury in deep-sea sharks of

the Southeastern Mediterranean Sea over the last ~ 40 years. Environ. Int. 187, 108661. https://doi.org/10.1016/j.envint.2024.108661

Souza, A. De, Vannuci-Silva, M., Rodrigues, L., Amaral, P., Felipe, H., Andrade, H., Azevedo, A.F., Malm, O., Lailson-Brito, J., Bisi, T.L., 2025. The tastiest is not always the safest: mercury risks of boneless fish consumption compared to other seafood traded in southeastern Brazil. Food Chem. Toxicol. 206. https://doi.org/10.1016/j.fct.2025.115729

Spanu, D., Butti, L., Boldrocchi, G., Bettinetti, R. & Monticelli, D. High-throughput, multi-batch system for the efficient microwave digestion of biological samples. Analytical Sciences 36, (2020).

Spanu, D., Butti, L., Recchia, S., Dossi, C. & Monticelli, D. Talanta A high-throughput , straightforward procedure for biomonitoring organomercury species in human hair. Talanta 270, 125612 (2024).

Storelli, A., Barone, G., Garofalo, R., Busco, A., Storelli, M.M., 2022. Determination of mercury, methylmercury and selenium concentrations in elasmobranch meat: fish consumption safety. Int. J. Environ. Res. Public Health 19. https://doi.org/10.3390/ijerph19020788

Storelli, M.M., Busco, V.P., Marcotrigiano, G.O., 2005. Mercury and arsenic speciation in the muscle tissue of *Scyliorhinus canicula* from the Mediterranean Sea. Bull. Environ. Contam. Toxicol. 75, 81–88. https://doi.org/10.1007/s00128-005-0721-0

Storelli, M.M., Ceci, E., Storelli, A., Marcotrigiano, G.O., 2003. Polychlorinated biphenyl, heavy metal and methylmercury residues in hammerhead sharks: contaminant status and assessment. Mar. Pollut. Bull. 46, 1035–1039. https://doi.org/10.1016/S0025-326X(03)00119-X

Storelli, M.M., Cuttone, G., Marcotrigiano, G.O., 2011. Distribution of trace elements in the tissues of smooth hound *Mustelus mustelus* (*Linnaeus*, 1758) from the southern – eastern waters of Mediterranean Sea (Italy). Environ. Monit. Assess. 174, 271–281. https://doi.org/10.1007/s10661-010-1456-x

Storelli, M.M., Giacominelli-Stuffler, R., Marcotrigiano, G., 2002. Mercury accumulation and speciation in muscle tissue of different species of sharks from Mediterranean Sea, Italy. Bull. Environ. Contam. Toxicol. 68, 201–210. https://doi.org/10.1007/s00128-001-0239-z

Suk, S.H., Smith, S.E., Ramon, D.A., 2009. Bioaccumulation of mercury in pelagic sharks from the Northeast Pacific Ocean. Calif. Coop. Ocean. Fish. Investig. Reports 50.

Taylor, D.L., Kutil, N.J., Malek, A.J., Collie, J.S., 2014. Mercury bioaccumulation in cartilaginous fishes from Southern New England coastal waters: Contamination from a trophic ecology and human health perspective. Mar. Environ. Res. 99, 20–33. https://doi.org/10.1016/j.marenvres.2014.05.009

Teffer, A.K., Staudinger, M.D., Taylor, D.L., Juanes, F., 2014. Trophic influences on mercury accumulation in top pelagic predators from offshore New England waters of the

northwest Atlantic Ocean. Mar. Environ. Res. 101, 124–134. https://doi.org/10.1016/j.marenvres.2014.09.008

Torres-Escribano, S., Ruiz, A., Barrios, L., Vélez, D., Montoro, R., 2011. Influence of mercury bioaccessibility on exposure assessment associated with consumption of cooked predatory fish in Spain. J. Sci. Food Agric. 91, 981–986. https://doi.org/10.1002/jsfa.4241

Torres, P., Tristão, R., Maia, R., Rodrigues, S., 2014. Trophic ecology and bioindicator potential of the North Atlantic tope shark. Sci. Total Environ. 481, 574–581. https://doi.org/10.1016/j.scitotenv.2014.02.091

Vélez-Alavez, M., Labrada-martagón, V., Méndez-Rodríguez, L.C., Galván-magaña, F., Zenteno-Savín, T., 2013. Comparative biochemistry and physiology, part a oxidative stress indicators and trace element concentrations in tissues of mako shark (*Isurus oxyrinchus*). Comp. Biochem. Physiol. Part A 165, 508–514. https://doi.org/10.1016/j.cbpa.2013.03.006

Vélez, N., Bessudo, S., Barragan-Barrera, D.C., Ladino, F., Bustamante, P., Luna-Acosta, A., 2021. Mercury concentrations and trophic relations in sharks of the Pacific Ocean of Colombia. Mar. Pollut. Bull. 173. https://doi.org/10.1016/j.marpolbul.2021.113109

Walker, C.J., Gelsleichter, J., Adams, D.H., Manire, C.A., 2014. Evaluation of the use of metallothionein as a biomarker for detecting physiological responses to mercury exposure in the bonnethead, *Sphyrna tiburo*. Fish Physiol. Biochem. 40, 1361–1371. https://doi.org/10.1007/s10695-014-9930-y

Walker, T.I., 1988. Mercury concentrations in edible tissues of elasmobranchs, teleosts, crustaceans and molluscs from south-eastern Australian waters. Aust. J. Mar. Freshw. Res. 39, 39–49. https://doi.org/10.1071/MF9880039

Wang, M., Chen, C., Paolo, F., Albarico, J.B., Tsai, W., 2023. Mercury and selenium concentrations and their toxicological implications in silky sharks *Carcharhinus falciformis* (Elasmobranchii: Chondrichthyes) in the northwestern Indian Ocean. Reg. Stud. Mar. Sci. 66, 103165. https://doi.org/10.1016/j.rsma.2023.103165

Wosnick, N., Paula, A., Daldin, R., Luiz, J., Nunes, S., Dillenburg, T., Pierre, S., Quental, I., Hauser-Davis, R.A., 2021. Nurse sharks, space rockets and cargo ships: Metals and oxidative stress in a benthic, resident and large-sized mesopredator, *Ginglymostoma cirratum*. Environ. Pollut. 288, 117784. https://doi.org/10.1016/j.envpol.2021.117784

Zaera, D., Johnsen, E., 2011. Foetal deformities in a smooth-hound shark, *Mustelus mustelus*, from an oil exploited area in Angola. Cybium 35, 231–236. https://doi.org/10.26028/cybium/2011-353-007
